# Microautophagy: current understanding of its molecular mechanisms and functions

**DOI:** 10.1080/27694127.2026.2626661

**Published:** 2026-02-24

**Authors:** Yasuyoshi Sakai, Christian Behrends, Jayanta Debnath, Masanori Izumi, Andreas Jenny, Maurizio Molinari, Shuhei Nakamura, Masahide Oku, Marisa S. Otegui, Laura Santambrogio, Han-Ming Shen, Tomohiko Taguchi, Michael Thumm, Takashi Ushimaru, Zhiping Xie, Ana Maria Cuervo, Fulvio Reggiori

**Affiliations:** a Graduate School of Advanced Integrated Studies in Human Sustainability, Kyoto University, Kyoto, Japan; b Department of Applied Biological Sciences, Faculty of Bioenvironmental Sciences, Kyoto University of Advanced Science, Kameoka, Kyoto, Japan; c Munich Cluster for Systems Neurology (SyNergy), Faculty of Medicine, Ludwig-Maximilians-Universität München, Munich, Germany; d Department of Pathology, University of California San Francisco, San Francisco, California, USA; e RIKEN Center for Sustainable Resource Science (CSRS), Wako, Japan; f Department of Developmental and Molecular Biology, Albert Einstein College of Medicine, New York, NY, USA; g Institute for Research in Biomedicine, Università della Svizzera italiana, Bellinzona, Switzerland; h School of Life Sciences, École Polytechnique Fédérale de Lausanne, Lausanne, Switzerland; i Department of Biochemistry, Nara Medical University, Kashihara, Nara, Japan; j Department of Botany and Center for Quantitative Cell Imaging, University of Wisconsin-Madison, Madison, WI, USA; k Weill Cornell Medicine, New York, NY, USA; l Faculty of Health Sciences, Ministry of Education Frontiers Science Center for Precision Oncology, University of Macau, Macau, China; m Laboratory of Organelle Pathophysiology, Department of Integrative Life Sciences, Graduate School of Life Sciences, Tohoku University, Sendai, Japan; n Department of Cellular Biochemistry, University Medical Center Gottingen, Göttingen, Germany; o Course of Biological Science, Department of Science, Graduate School of Integrated Science and Technology, Shizuoka University, Shizuoka, Japan; p School of Life Sciences and Biotechnology, Shanghai Jiao Tong University, Shanghai, China; q Department of Biomedicine, Aarhus University, Aarhus C, Denmark

**Keywords:** Endosomes, lysosomes, multivesicular bodies, organelle turnover, proteolysis, vacuole

## Abstract

Microautophagy (MI-autophagy) is an umbrella term for intracellular degradative pathways that entail the invagination or protrusion of the limiting membranes of endolysosomal compartments, that is, late endosomes and mammalian lysosomes or yeast and plant vacuoles, followed by pinching-off of the membrane into the lumen of the organelle. During these processes, the material specifically and nonspecifically targeted for degradation is sequestered within the invaginating or protuberating membrane. In contrast to macroautophagy, the molecular mechanisms underlying MI-autophagy are largely unknown due to their diversity and complexity in location, regulation and molecular machinery requirements. Here, we review recent progress in the field of MI-autophagy, describing the molecular basis and functions of the MI-autophagic pathways reported to date in eukaryotic cells, from yeast to mammalian and plant cells.

## Introduction

The term autophagy defines intracellular transport systems for the delivery and degradation of intracellular components, including protein aggregates, organelles and pathogens, into endosomes, vacuoles or lysosomes. These pathways play multiple physiological roles such as protein quality control, metabolism, cell differentiation and immunity, among others, and their defect underlies the basis of various pathologies. According to the membrane dynamics characterizing how the cargo is transported into the degradative organelle (DO), autophagy was classified into macroautophagy and microautophagy (MI-autophagy) by De Duve and Wattiaux (1966) [1]. During macroautophagy, the cargoes are sequestered by newly generated cisternae called the phagophores, also known as isolation membranes, which close into double-membrane vesicles called autophagosomes. Autophagosomes can directly fuse with lysosomes, to form autolysosomes, or can first fuse with late endosomes to form amphisomes that then fuse with lysosomes. During MI-autophagy, in contrast, lysosomes or endosomes directly engulf the cargo via the inward budding or a protrusion of their limiting membrane to incorporate the material targeted for degradation [2–9]. Subsequently, a third type of autophagy, chaperone mediated autophagy (CMA), was discovered, in which single proteins carrying a KFERQ-like motif are directly translocated into the lysosome interior through a translocation complex formed by multimerization of LAMP2A (lysosomal associated membrane protein 2A) [10]. While macroautophagy and MI-autophagy are present in all eucaryotes, from yeast to mammals and plants, CMA is only found in mammalian cells [6,11].

Our understanding of MI-autophagy remained mostly descriptive until studies in yeast in the 1990s that identified MI-pexophagy in the methylotrophic yeast *Komagataella phaffii* (also known as *Pichia pastoris*) [12,13]. Subsequent studies revealed that some of the ATG (autophagy related) proteins that are key components of macroautophagy also function in MI-pexophagy at the vacuole (v-MI-pexophagy) [14,15]. Later work in yeast and plant cells uncovered that vacuolar microautophagy (v-MI) also targets other organelles, including portion of the nucleus [16], endoplasmic reticulum (ER) [17], lipid droplets (LDs) [18,19], mitochondria [20], and chloroplasts [21].

While the involvement of Atg proteins has been reported in many of these selective types of v-MI [14,18,22], some of these pathways also require components of the endosomal sorting complex required for transport (ESCRT) [19,23]. Thus, the current view is that ATG and/or ESCRT proteins are key players in v-MI [6]. However, v-MI processes that do not depend on ATG and/or ESCRT proteins cannot be excluded (Table 1).

**Table 1.** Molecular Dependence and Membrane Dynamics of v-MI and l-MI Pathways.*

MI-autophagy	Membrane deformity	Core ATG/Atg proteins	ESCRT	Other specific molecules
ATG-dependent v-MI-pexophagy	Type 1	Atg1, Atg2, Atg3, Atg4, Atg5, Atg7, Atg8, Atg9, Atg11, Atg12, Atg14, Atg16, Atg18	**	Atg21, Snx4/Atg24, Atg25, Atg26, Atg27, Atg28, Atg30, Vac8, Vam7, Vps34, Vps15, Trs85, Pik1, Pfk1, Gcn1, Gcn2, Gcn3, Gcn4
ESCRT-dependent v-MI-lipophagy	Type 2		Vps27***, Vps23, Vps36, Snf7, Vps4	Ldo16, Ldo45, Trs85, Vam3, Vam7, Vac8, Ypt7
ATG-dependent v-MI-lipophagy	Type 2	Atg1, Atg2, Atg3. Atg4, Atg5, Atg6, Atg7, Atg8, Atg9, Atg10, Atg12, Atg13, Atg14, Atg16, Atg17, Atg18, Atg21, Atg22, Atg23, Atg29, Atg31, Atg32, Atg34		
v-MI-nucleophagy	Type 1	Atg1, Atg2, Atg3, Atg4, Atg5, Vps30/Atg6, Atg7, Atg8, Atg9, Atg10, Atg11, Atg12, Atg13, Atg14, Atg15, Atg16, Atg17, Atg18, Atg29, Atg31	Vps27, Vps28, Vps36, Vps24, Vps4, Chm7	Nvj1, Vac8, Scs2, Scs22, Trs85, Ypt7, Vam3, Vam7
ESCRT-dependent v-MI-ERphagy	Type 1		Vps27, Hse1, Vps23, Vps37, Mvb12, Vps22, Vps28, Vps36, Vps25, Vps20, Snf7,Vps24, Vps2, Vps4 Bro1	Nem1-Spo7 complex (Nem1, Spo7, Pah1)
ATG-depemdent v-MI-chlorophagy	Type 2	ATG4, ATG5, ATG7		
ATG-independent v-MI-chlorophagy	Type 2			NBR1
ESCRT-dependent v-MI-vacuolophagy	Type 2	Atg8	Vps27, Vps23, Vps36, Snf7, Vps4	Hfl1
l-MI-mitophagy	Type 2			SQSTM1/p62, TOLLIP, RHOT1/MIRO1, DNM1L/DRP1, PRKN, RAB32, RAB38
ESCRT-dependent l-MI-lysophagy	Type 2		CHIMP4A, CHIMP4B, VPS4A	RNF152, LAPTM4A, NEDD4
ATG and ESCRT-dependent l-MI-lysophagy	Type 2	ATG3, ATG7, ATG8s (GABARAPs), ATG16L1	Tsg101, PDCD6IP/ALIX, CHIMP4A, CHIMP4B, VPS4A, VPS4B	STK38, DOK1

*Blank columns: The molecular requirement has been unpublished, and is open for future research.

**Recent data supported requirement of ESCRTs including Vps27 (Sakai & Oku, unpublished).

***Requirement of Vps27 depends on MI-lipophagy-inducing conditions.

Thereafter, endosomal microautophagy (e-MI) was also identified in mammalian cells, fly neurons, and the fission yeast *Schizosaccharomyces pombe*, and it was shown to depend on the function of the ESCRT system and in several instances also of the HSPA8/HSC70 (heat shock protein family A (Hsp70) member 8) chaperone, which recognizes the same KFERQ-like motif previously identified for CMA (Table 2) [24–28]. Very recent studies have also uncovered MI-autophagy by lysosomes, i.e., lysosomal MI-autophagy (l-MI), in mammals [8]. Notably, some of the l-MI pathways also employ ATG proteins, namely those belonging to the machinery conjugating Atg8-protein family members mainly to phosphatidylethanolamine (PE) (Table 1) [29,30].

**Table 2.** Comparison of the Different Types of e-MI in Mammals and Flies.

			Mammals						Drosophila	
			In bulk	HSC70- mediated	Starvation-induced	TOLLIP-mediated	e-MI- aggrephagy	e-MI-ERphagy*	Hsc70- mediated	In bulk
Organelle			LE/MVB	LE/MVB	LE/MVB	EE, LE/MVB	LE/MVB	LE/MVB	LE/MVB	LE/MVB
Membrane Deformity			Type 2	Type 2	Type 2	Type 2	Type 2	Type 2	Type 2	Type 2
ESCRT		Required	TSG10, PDCD6IP/ALIX, VPS4A/B	TSG101, PDCD6IP/ALIX, VPS4A/B	CHMP4B, VPS4A/B	CHMP4B	TSG101, VPS4A/B, VPS37A, ABAP1, VAPS28, PTPM23, CHIMP1A	TSG101, VPS4A/B		Vsp25, Vsp28, Vsp32
		Dispensable			TSG101, VPS28, SNF8/VPS22/EAP30, CHMP3/VPS24, CHMP4A/C		VPS37B, STAM1/2, VPS22, VPS25, VPS36, CHMP4B, CHMP5, CHMP6			Hgs/Hrs, Stam (partially)
ATG		Required	–	–						Atg1, Atg13
		Dispensable	ATG5, ATG7, BECN1	ATG5, ATG7, BECN1	PIK3C3/VPS34, ULK1/2, ATG7, STX17	PIK3C3/VPS34, ATG14, ULK1/2	ATG7, RB1CC1/FIP200			Atg5, Atg7, Atg12
Targeting tag			No	KFERQ	No	Ubiquitin	Ubiquitin	K63-ubiquitination on STING1	KFERQ	No
Receptor/Adaptor			No	HSPA8/ HSC70	Unknown	TOLLIP	TSG101		Hsc70-4	Hsc70-4
Other components				BAG6						Csp/DNACJ5
Stimulus	Starvation		No changes	Inhibits	Induces				Induces	Induces
	DNA damage			Induces					Induces	Induces
	ROS									Induces
	ER stress									no change

*Some forms of ER MI-autophagy may occur in lysosomes instead of LE/MVB, but the specific requirements remain unknown.

**Figure 1.**
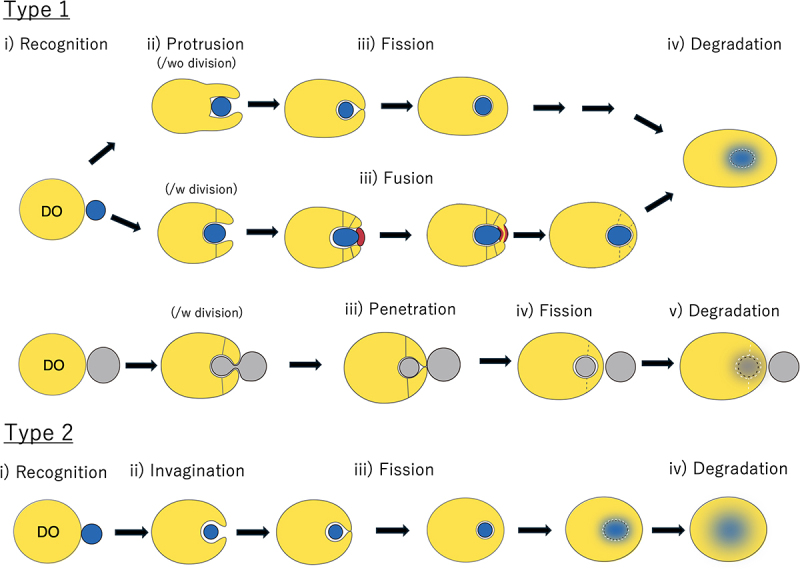
Membrane dynamics and classification of MI-autophagic pathways in type 1 and type 2. (i) All the processes start with recognition of the cargo by the degradative organelle (DO) (yellow). (ii) dynamics of the DO during the capture of the cargo (blue or gray). *type 1*: the DO extends a membranous arm by protrusion (upper sequence). In v-MI-pexophagy and v-MI-nucleophagy, the DO protrudes in concomitance with its division (middle sequence). In v-MI-nucleophagy and certain MI-ERphagy pathways, the DO membrane penetrates within the organelle targeted for degradation, an event followed by membrane fission to release the rest of the organelle (lower sequence). *type 2*: the limiting membrane of the DO invaginates during the capture of the cargo. The recognition of the substrate results in protrusion of the DO around the cargo. (iii) sequestration of the cargo is completed by fission of the lytic organelle membrane (fission type) or fusion of the organelle shown in red color (fusion type). (iv) the MI-autophagic body and its cargo are degraded.

Because of this complex scenario, we recently proposed a more systematic nomenclature to distinguish the diverse MI-autophagic pathways based on their cargo and the location where MI-autophagy occurs, such as the vacuole (v-MI), lysosomes (l-MI) and endosomes (e-MI) [31] (please, note that in this review, we have reserved the term endolysosome for those instances in which studies attempting to identify if the DO is a late endosome or a lysosome are still not available). We further classified MI-autophagic processes into type 1 and type 2 depending on the membrane dynamics how the DO sequesters the cargo destined to degradation (Figure 1) [31]. Type 1 involves extension, often accompanied by division, or protrusion of the limiting membrane of the vacuolar or lysosomal membrane, followed by its scission. There are so far no reports suggesting that Type1 MI-autophagy occurs at endosomes. In contrast, the process is classified as type 2 when the targeted component is directly engulfed by invagination of the DO’s limiting membrane [31].

Here, we provide an updated review about recent progress in our understanding of the mechanisms and functions of the diverse MI-autophagic pathways, highlighting differences and common requirements, which underscore the existence of parallel processes regulated by distinct molecular players and sometime also characterized by morphologically distinct membrane rearrangements.

## v-MI-autophagy pathways in yeast and plants

### v-MI-pexophagy

The peroxisome is a vital organelle for various metabolic processes, such as beta-oxidation of fatty acids or plasmalogen synthesis [32]. Autophagic degradation of peroxisomes, i.e., pexophagy, constitutes the main system to regulate the quantity of these organelles, which coincides with a reduction of the peroxisome-related metabolic activities [33]. In most organisms, pexophagy is carried out by selective macroautophagy [34]. *K. phaffii* is a unique organism since after augmentation of the number and size of peroxisomes, their degradation via pexophagy takes place through either MI-autophagy or macroautophagy, depending on nutrient conditions [13]. In this organism, the MI-autophagic pathway, i.e., v-MI-pexophagy, has been morphologically characterized mostly using fluorescence microscopy [12,14], revealing that peroxisomes are engulfed by extensions of the vacuolar membrane characteristic of type 1 MI-autophagy [4,31]. The formation of this extension, which often accompanied septations at the edge of the extension, was later found to depend on Atg18 [35,36] and Vac8 [37]. Another study suggested that Atg24, a phosphatidylinositol-3-phosphate (PtdIns3P)-binding protein, regulates the vacuolar protrusion and its fusion during v-MI-pexophagy [38].

Extensive studies have identified the molecular mechanisms of MI-pexophagy in *K. phaffii*. One remarkable finding is the requirement of the core Atg proteins for this process (Table 1) [39]. Morphological analyses revealed the formation of phagophore-like structures adjacent to the target peroxisomes [40]. This structure, which bridges the edges of the vacuolar membrane extension and was named micropexophagic membrane apparatus (MIPA), is formed by the Atg proteins and labeled with lipidated Atg8 (Figure 1). MIPA leads to the complete sequestration of the target peroxisomes from the cytoplasm. Hence, MI-pexophagy in this organism involves both vacuolar membrane dynamics and the Atg-dependent formation of MIPA.

**Figure 2.**
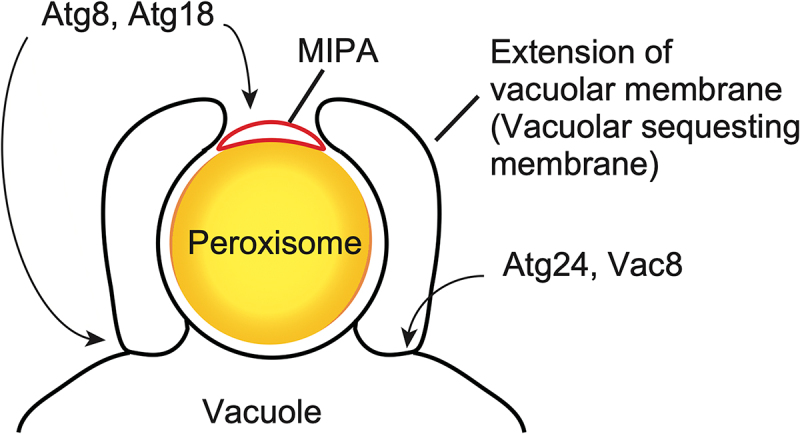
Membrane structures and key factors involved in *K. phaffii* v-MI-pexophagy. The target peroxisomes are engulfed by extension of the vacuolar membrane. This extension often accompanies the fission of the vacuolar membrane occurring at the Atg24- and Vac8-enriched septa. The phagophore-like structure termed MIPA, is formed by the actions of core Atg proteins. Atg8 and Atg18 are dually localized, i.e., to the septa and the MIPA, and regulates the formation and morphology of the membranous structures mediating v-MI-pexophagy.

Several Atg proteins act on two distinct locations during MI-pexophagy, i.e., the vacuolar membrane and the MIPA (Figure 2). Atg8, a hallmark protein of autophagic membranes, localizes to the MIPA as described above as well as to the vacuolar membrane, where it is important to regulate the morphology of this organelle. Notably, while lipidation is dispensable for Atg8 association with the vacuolar membrane, cleavage of Atg8 at the C terminus is essential for its function on the vacuolar membrane [41]. Atg18 also localizes to both the vacuolar membrane and MIPA and has a dual role in the v-MI-pexophagy, i.e., the generation of MIPA, and the septation and protrusion of the vacuolar membrane. The association of Atg18 with the vacuolar membrane during v-MI-pexophagy is regulated by phosphorylation at a serine residue of Atg18 in the close vicinity to its characteristic beta propeller structure. Specifically, phosphorylation of Atg18 decreases its affinity for PtdIns3P and PtdIns(3,5)P_2_, two phosphoinositides present in the vacuolar membrane that mediate Atg18 recruitment[36]. To date, whether v-MI-pexophagy utilizes components of the ESCRT pathway remains an open question.

### v-MI-lipophagy

Since the internal contents of LDs, i.e., triglycerides and sterol esters, function as the reservoir of intracellular fatty acids and sterols, the quantitative regulation of this organelle is a crucial factor for maintaining the cellular lipid homeostasis [42]. Early studies focused on the biosynthetic and degradation processes of neutral lipids by enzymes resident on the surface of LDs [43]. Later, macroautophagy emerged as another important regulatory mechanism of the cellular content of lipids, through what was termed lipophagy [44]. Yet, it should be emphasized that the autophagic clearance of the organelle also leads to the degradation of LD proteins, which is likely to have other physiological consequences.

In the yeast *S. cerevisiae*, the vacuolar MI-autophagic turnover of LDs (v-MI-lipophagy) through a type 2 mechanism has been observed under multiple environmental conditions, while no macroautophagic degradation of the organelle has been reported so far. It has been shown that stimuli triggered by nutrient deprivation, such as nitrogen starvation [18] and glucose starvation [45], induce v-MI-lipophagy. Growth phase transition to stationary phase [46], diauxic shift [19], lipid imbalance and ER stress [47,48] also upregulate v-MI-lipophagy in this organism. The findings from different studies on v-MI-lipophagy are difficult to be reconciliated into a single model, leading to the idea that several diverse mechanisms drive v-MI-lipophagy.

Recent studies described a type of v-MI-lipophagy that does not depend on the Atg proteins but instead requires ESCRT proteins (Table 1) [19,47,48]. The diauxic shift- and ER stress-induced v-MI-lipophagy required the ESCRT machinery, and a component of ESCRT-0, Vps27 (vacuolar protein sorting 27), was observed to be translocated onto the vacuolar membrane during diauxic shift, suggesting the direct action of the ESCRT machinery on the vacuolar membrane mediates this type of MI-lipophagy [19]. One study, in particular, reported an inhibitory effect of the ESCRT components Vps27 or Snf7 on v-MI-lipophagy induced by glucose restriction, which implies different functions of the ESCRT system in MI-lipophagy [49].

A group of antecedent studies, however, indicated the functional involvements of the core Atg proteins in v-MI-lipophagy when this process is induced as macroautophagy by nutrients starvation or entrance into stationary phase (Table 1) [18,45,46,50]. In these situations, no phagophores or autophagosome-like vesicle sequestering the LDs were detected, making unlikely that the Atg proteins function in this type of v-MI-lipophagy is the *de novo* formation of a membranous structure that selectively target LDs. A more plausible explanation is that these Atg proteins are responsible for generating microdomains within the vacuolar membrane, which mediates the direct engulfment of the targeted LDs by the vacuolar membrane. Along this line, the association of a subset of Niemann-Pick type C proteins in the vacuole was found to depend on Atg proteins, which may explain the functional involvements of the Atg proteins in the lipid microdomain formation in which sterol plays a pivotal role [50]. Thus, the molecular requirement of Atg proteins possibly reflects an indirect contribution of macroautophagy to MI-lipophagy.

### v-MI-nucleophagy

In *S. cerevisiae*, v-MI-nucleophagy, also known as piecemeal MI-autophagy of the nucleus (PMN), takes place at the nucleus-vacuolar junctions (NVJs) [16,51]. The interaction of the nuclear ER membrane protein Nvj1 with the vacuolar protein Vac8 is the basis for the generation of NVJs. Upon nitrogen starvation or addition of rapamycin, the NVJ bulges out into the vacuole. Most likely, the next step involves a micronucleus containing non-essential parts of the nucleus, in which the nucleolus buds off within the vacuolar invagination. Finally, a v-MI-nucleophagic body is released into the vacuole for degradation. Accordingly, the resulting MI-nucleophagic body is surrounded by three membranes; from the outside to the inside, these include the vacuolar membrane, the outer and inner nuclear membrane. Thus, v-MI-nucleophagy is a type 1 process.

v-MI-nucleophagy depends on the core Atg machinery (Table 1) [22] and microscopic analyses have suggested the formation of a MIPA-like structure, similar to the one described for v-MI-pexophagy, between the vacuolar edges [52]. This indicates a possible role of the Atg machinery in sealing the vacuolar edges during the formation of the v-MI-nucleophagic body. Additionally, the ESCRT system is involved in v-MI-nucleophagy induced by rapamycin treatment (Table 1) [53,54].

Nucleolar proteins, including the subunits of the immature ribosome precursors, are not essential under nutrient-starved conditions, where ribosome biogenesis is repressed. Elimination of these nucleolar proteins by v-MI-nucleophagy may be important for starvation-adapted nuclear reconstruction. In contrast, chromosomes are still essential in starved cells and consequently selective degradation of nucleolar proteins without affecting chromosomes is vital. Nucleolar proteins move toward the NVJ, where v-MI-nucleophagy occurs, whereas rDNA, which is embedded in nucleolar proteins under normal (nutrient-rich) conditions, is segregated from nucleolar proteins to escape from NVJs during nutrient starvation [55]. This nucleolar remodeling is driven by rDNA condensation [56] and it is associated with v-MI-nucleophagy in an NVJ-dependent manner [57]. This demonstrates that vacuoles control the remodeling of the intranuclear compartment via the NVJs, but the underlying molecular mechanisms are currently unknown.

NVJ integrity is critical for v-MI-nucleophagy, but not for macronucleophagy. That is, mutant yeast cells lacking NVJs abolish MI-nucleophagic degradation of nucleolar proteins while retaining macronucleophagic activity [57]. This indicates that macroautophagy is independent of v-MI-nucleophagy. In contrast, v-MI-autophagic flux depends on macroautophagy, which degrades Nvj1, a critical v-MI-nucleophagy factor [58].

### v-MI-ERphagy

The endoplasmic reticulum (ER) is the organelle of eukaryotic cells deputed for the synthesis of proteins, lipids and sugars, and their sorting to other subcellular locations. The ER activities and size are enhanced upon activation of the transcriptional and translational programs named unfolded protein responses (UPR), first reported by pioneers of electron microscopy in the late 1950s (e.g., [59,60]). And endoplasmic reticulophagy (ERphagy) is now understood mechanistically in detail in both yeasts and mammals [61].

In yeast, the vacuole membrane buds inward to capture the ER whorl extensions caused by high-level expression of ER-resident membrane proteins, in a v-MI-ERphagy process that relies on the ESCRT I-III machinery and the Nem1-Spo7 phosphatase complex (Table 1) [23,62].

### v-MI-chlorophagy

All plant cells contain plastids, which differentiate into chloroplasts and serve as the site for photosynthesis in green tissues such as those in leaves. Chloroplasts contain a closed internal membrane system called thylakoids, where light-harvesting complexes and an electron transport chain convert light into chemical energy. Carbon dioxide assimilation and carbohydrate synthesis occur in the chloroplast stroma, the matrix surrounding the thylakoids. During photosynthesis, sunlight is used to break apart water molecules, releasing oxygen gas, hydrogen ions, and electrons. This causes chloroplasts to be constantly exposed to oxidative damage, even more so under high light conditions. Photodamage drastically reduces photosynthetic rates, limiting plant growth[63]. Therefore, the ability to repair and remodel photodamaged chloroplasts plays a fundamental role in plant growth and stress tolerance. Consistently, plants employ several macroautophagy and MI-autophagy pathways to remove parts of or whole damaged chloroplasts. These pathways are known as macrochlorophagy and MI-chlorophagy.

At least two MI-chlorophagy pathways have been reported in plants, one dependent on the canonical ATG machinery and the other one, independent from it (Table 1). *Arabidopsis thaliana* mutants lacking core ATG proteins show enhanced sensitivity to damage caused by ultraviolet-B (UVB) irradiation [64,65]. Microscopy observations revealed that a subpopulation of photodamaged chloroplasts is transported into the vacuolar lumen via v-MI-chlorophagy when the mature leaves are exposed to UVB or intense visible light [64]. This vacuolar delivery does not occur in the *atg5* or *atg7* mutant plants [64], indicating that this MI-chlorophagy pathway requires the canonical ATG machinery. Additional *in vivo* imaging demonstrated that swollen chloroplasts appear after exposure to strong visible light and become associated with partial coats labeled by GFP-ATG8. Finally, these chloroplasts are engulfed by the vacuolar membranes [21]. Since chloroplasts also accumulate in the vacuolar lumen of senescing *Arabidopsis* leaves from wild-type (WT) plants but was not from the *atg4* mutant [66], suggesting that canonical v-MI-chlorophagy may also function during leaf aging.

The current knowledge about molecular basis underlying canonical v-MI-chlorophagy is limited. Plant E3 ligases PUB4 (plant U-box4) and SP1 (suppressor of ppi1 locus) individually mediate the ubiquitination of chloroplast proteins in response to oxidative damage [67–69]. Mutations in each E3 ligase does not alter the activity of canonical v-MI-chlorophagy [70], indicating that ubiquitination by those E3 ligases is not required for this pathway. The partial ATG8-positive coats that associate with damaged chloroplasts could be mediating membrane fusion at the final step of v-MI-chlorophagy to completely sequester the targeted chloroplasts. These coats may be similar to the MIPA, which partially coats peroxisomes destined to MI-pexophagy in methylotrophic yeasts as above-described [40]. Another possibility is that ATG8 binds to SARs or other adaptor proteins on damaged chloroplasts to facilitate canonical MI-chlorophagy.

Interestingly, another v-MI-chlorophagy pathway operates even when the canonical ATG machinery is blocked. A recent study found that NBR1 (NBR1 autophagy cargo receptor) specifically associates with a subset of photodamaged chloroplasts that are engulfed by vacuoles through v-MI-chlorophagy in *Arabidopsis* leaves [71]. The chloroplast population labeled by NBR1 is different from that labeled by ATG8 and the association of NBR1 with photodamaged chloroplast does not depend on the core ATG machinery, indicating that the ATG-dependent and ATG-independent v-MI-chlorophagy pathways are largely independent from each other [71]. The recruitment of NBR1 to damaged chloroplasts depends on the ability of NBR1 to bind ubiquitin. Photodamaged chloroplasts often lose envelope integrity. Thus, the cytosolic ubiquitination machinery can access the stroma and thylakoid of partially broken chloroplasts, leading to protein ubiquitination and recruitment of NBR1. NBR1-coated chloroplasts are then directly engulphed by vacuoles. Both pathways, i.e., ATG-dependent and ATG-independent v-MI-chlorophagy, are activated under photodamaging conditions [21,71], supporting a partially redundant and robust mechanism for the elimination of damaged chloroplasts to sustain photosynthetic activity. Both these v-MI-chlorophagy pathways are type 2 MI-autophagy (Table 1).

A recent study has reported that NBR1 targets specific ubiquitinated proteins in chloroplast outer-envelope membranes via macrochlorophagy [72]. The translocon at the outer envelope membrane of chloroplasts (TOC) is an import channel for nuclear-encoded chloroplast proteins. NBR1 recognizes K63-polyubiquitinated TOC subunits and targets them to autophagosomal delivery into the vacuole under UVB or heat stress conditions [72]. This process does not occur in the *atg7* mutants. Thus, NBR1 plays roles in both ATG-independent v-MI-chlorophagy and ATG-dependent macrochlorophagy under abiotic stress conditions. How these roles of NBR1 in chlorophagy are regulated or coordinated remains to be elucidated. Thus, there are several v-MI-chlorophagy pathways that coordinate the turnover and remodeling of chloroplasts under specific environmental changes.

## v-MI-vacuolophagy

### Yeast

MI-autophagy can mediate the turnover of vacuolar membrane proteins in a process called v-MI-vacuolophagy. In principle, it is plausible that some vacuolar membrane proteins are internalized during various v-MI-autophagy processes, as the internalization of other cytoplasmic cargos is always accompanied by the internalization of a portion of the vacuolar membrane. Conversely, v-MI-vacuolophagy is likely accompanied by the internalization of some cytoplasmic components, which has indeed been demonstrated [73–75]. Although the term v-MI-vacuolophagy emphasizes the vacuolar membrane substrates, it does not exclude the concurrent presence of cytosolic cargos.

Depending on the inducing condition, v-MI-vacuolophagy can display varying degrees of selectivity. Many vacuolar membrane proteins are nutrient transporters and respond to changes in the availability of the nutrients they transport. For instance, lysine deprivation triggers selective v-MI-autophagy of Ypq1 (yeast PQ-loop protein 1), a cationic amino acid transporter [76]. Similarly, manipulation of zinc levels triggers the turnover of zinc transporters Cot1 (cobalt toxicity 1) and Zrt3 (zinc-regulated transporter 3) [77]. Under other stress conditions, the turnover can be much broader. Several studies have investigated the transition from a nutrient-rich, logarithmic growth phase to a nutrient-depleted stationary phase, finding that under such conditions most vacuolar membrane proteins tested underwent v-MI-autophagic turnover [19,73,78]. Unlike acute experimental depletion of a single nutrient, the entry into stationary phase is a gradual process spanning dozens of hours, encompassing the diauxic shift (from glucose fermentation to ethanol oxidation). This v-MI-vacuolophagy-inducing condition has been described as post-diauxic shift, natural starvation, or early stationary phase. Diverse proteins have been confirmed to undergo v-MI-vacuolophagy-dependent turnover, including Cot1, Dpp1 (diacylglycerol pyrophosphate phosphatase 1), Fet5 (ferrous transport 5), Fth1 (FTR1 Homolog 1), Ncr1 (Niemann-Pick type C Related 1), Pho8 (phosphate metabolism 8), Vba4 (vacuolar basic amino acid transporter 4), Vph1 (vacuolar pH 1), Ypq1, Ypg2 (yeast PQ-loop protein 2), Ypl162c, and Zrt3 [73,78]. Among transmembrane proteins, only Ycf1 (yeast cadmium factor 1) and Zrc1 (zinc resistance conferring 3) are excluded from degradation. Interestingly, peripheral membrane proteins are also spared [73]. Ubiquitination plays a significant role in the turnover of v-MI-vacuolophagy substrates, triggered by changing nutritional conditions, regardless of whether the targets are a narrow or a broad number of proteins, and several E3 ligases have been identified [76–78]. Substrate-E3 ligase pairing is context-dependent, since a single substrate can be modified by different E3 ligases, depending on the inducing condition [77,78]. One exception is Pho8, whose targeting may potentially involve piggybacking instead of its own ubiquitination [73]. V-MI-vacuolophagy can also be induced by heat stress, although the level of selectivity is not well defined [74].

Mechanistically, all v-MI-vacuolophagy examples discussed above are type 2 and depend on the ESCRT machinery (Table 1) [19,73,74,76–79]. Most studies have observed defective turnover of v-MI-vacuolophagy in knockout mutants ablating the various ESCRT complexes. Considering the pleiotropic impacts of ESCRT knockouts, several studies have provided more direct evidence, using either a temperature-sensitive *vps4* allele or an auxin-inducible degron to acutely inactivate ESCRT, often in combination with a tet-off system to focus on preexisting vacuolar membrane proteins [73,78,80]. Under such conditions, preexisting vacuolar membrane proteins can be seen accumulating on the vacuolar membrane. Another line of evidence supporting the direct involvement of ESCRT is the observation that components of this system colocalize with substrate proteins on the vacuolar membrane [19,80]. However, such colocalization is not always prominent, likely because the association of ESCRT with the vacuolar membrane is transient. This technical limitation can be overcome by ATP depletion to arrest the transient ESCRT complexes [80]. The recruitment of ESCRT complexes relies on both substrate ubiquitination and local PtdIns3P production [19,81], analogous to the mechanism occurring at late endosomes/multivesicular bodies (LEs/MVBs). When v-MI-vacuolophagy is triggered by artificial ubiquitin tagging of a single substrate, the size of the generated lumenal vesicles is comparable to those in LEs/MVBs, i.e., around 40 nm in diameter [80]. In contrast, during v-MI-vacuolophagy occurring in early stationary phase, the vesicles are larger, around 300 nm in diameter [73]. The precise reason behind this size difference remains unclear, although the increase in vesicle size during early stationary phase may reflect the need to internalize a wide variety of substrates for energy generation.

Downstream of the ESCRT machinery, Atg8 has been identified as a key regulator of the membrane invagination process during early stationary phase (Table 1) [73]. This represents a v-MI-autophagy-specific function of Atg8 that is not shared by other Atg proteins such as Atg1, Atg2, Atg7, or Atg9. Mechanistically, Atg8 is recruited to the vacuolar membrane by the transmembrane protein Hfl1 (has fused lysosomes 1). As a result, the function of Atg8 does not require its lipidation. However, forced lipidation of Atg8 can bypass the need for Hfl1. Hfl1 contains seven predicted transmembrane domains and has been shown to regulate vacuole morphology together with Atg8 [82]. From the perspective of MI-vacuolophagy, Hfl1 can be considered a unique substrate, as its interaction with Atg8 serves to protect Hfl1 from internalization [73]. In the absence of Atg8, Hfl1 or their interaction, v-MI-vacuolophagy is kinetically slowed, accompanied by the accumulation of much larger lumenal vesicles up to 1 μm in diameter [73]. A role for Atg8 in regulating the size of lumenal vesicles has also been reported in heat-stress-induced v-MI-vacuolophagy, although the involvement of Hfl1 has yet to be tested in this case [79]. A recent study demonstrated that Atg8 has a membrane-perturbation activity [83], which may be responsible for the Atg8 involvement in a subset of v-MI-vacuolophagy pathways.

Proteins involved in vacuole-related membrane fusion, including the homotypic fusion and protein sorting (HOPS) tethering complex and soluble NSF attachment protein receptor (SNARE) proteins, also play a role in v-MI-vacuolophagy [73,80]. This genetic dependency appears to imply a link between v-MI-vacuolophagy and the intralumenal fragment pathway, wherein lumenal membrane pieces are generated as a byproduct of homotypic vacuole fusion [84]. However, v-MI-vacuolophagy differs from the intralumenal fragment pathway in two key aspects: the sequestration of cytosol [73,74], and the utilization of the ESCRT machinery. Additionally, it has been found that intralumenal fragment formation was not a major contributor to the turnover of vacuolar membrane proteins [85]. The dependence of v-MI-vacuolophagy on the fusion machinery can instead be explained by an indirect role in the trafficking of E3 ligase subunits, such as Ssh4 (suppressor of Shr3 deletion), and possibly other transmembrane proteins important for v-MI-vacuolophagy, to the vacuole [80].

The upstream signaling pathways controlling v-MI-vacuolophagy likely differ depending on the inducing conditions. As yeast cells approach nutrient-depleted stationary phase, inactivation of the target of rapamycin complex 1 (TORC1) is a key signaling event that leads to upregulation of ubiquitination [78]. Inactivation of TORC1 also eliminates an inhibitory phosphorylation of Vps27, a crucial subunit of the ESCRT-0 complex [86]. Pharmacological inactivating TORC1 with rapamycin can replicate many of these observed changes. The upstream signaling in most other conditions remains to be elucidated.

### Plants

Plant cells also employ MI-autophagy for vacuolar membrane degradation, v-MI-vacuolophagy. Portions of the vacuolar membrane are invaginated into the vacuolar lumen when tobacco cultured cells or *Arabidopsis* roots are subjected to energy deficit via sugar starvation, or when *Arabidopsis* roots are exposed to a high concentration of ammonium [87,88]. The lack of ATG2, ATG5 or ATG7 only partially suppresses vacuolar membrane invagination [87]. Therefore, it is unclear whether core ATG proteins directly regulate v-MI-vacuolophagy in plants.

## l-MI-autophagy pathways (mammal and *Caenorhabditis elegans*)

### l-MI-mitophagy

Mitochondria are dynamic organelles that carry out a multitude of functions, including the generation of the energy required to execute almost all cellular activities [89]. Mitochondrial homeostasis is maintained by canonical macromitophagy upon acute mitochondrial depolarization [90,91], as well as via mitochondria-derived vesicles directed to lysosomes or MI-mitophagy pathways that are triggered by milder damages as those induced by oxidative stress [92], or when macromitophagy is dysfunctional [91]. MI-mitophagy ensures the lysosomal clearance of mitochondrial-derived vesicles (MDVs) that selectively incorporate mitochondrial components that must be removed from the organelle [93,94]. For example, the generation of MDVs during yeast v-MI-mitophagy allows the removal of mitochondria subdomains containing aged transmembrane proteins of the inner and outer mitochondrial membranes[95]. In mammalian cells, two families of MDVs have been thoroughly studied, the TOMM20 (translocase of outer mitochondrial membrane 20)-positive MDVs [96,97] and the pyruvate dehydrogenase (PDH)-positive MDVs [98,99]. TOMM20-positive MDVs contain all the beta-barrel proteins and the subunits of the translocase of the outer mitochondrial membrane (TOMM) import complex and are generated from mitochondrial bulges initiated by the GTPases RHOT1/MIRO1 (ras homolog family member T1) and MIRO2, and their binding partners. Scission of the MDVs is controlled by the GTPase DNM1L/DRP1 (dynamin 1 like) [96]. Delivery of MDVs to degradative lysosomal compartments in response to mitochondrial stress additionally relies on the E3 ubiquitin ligase PRKN (parkin RBR E3 ubiquitin protein ligase) and the SAR TOLLIP (toll interacting protein) [97]. This process is evolutionary conserved and executed in yeast by Gem1 (GTPase EF-hand protein of mitochondria) and Dnm1, the orthologs of MIRO1 and DNM1L/DRP1, independent of Atg32, the key SAR for macromitophagy in yeast [100,101]. More recently, it has been revealed that in macrophages, lysosomes engulf various organelles, including mitochondria and endosomes through a type 2 mechanism that does not depend on ATGs [i.e., ATG7 and RB1CC1/FIP200 (RB1 inducible coiled-coil 1)] and ESCRT [i.e., TSG101 (tumor susceptibility 101), CHIMP3 and VSP4] machineries but requires the redundant RAB32 and RAB38 GTPases, PtdIns(3,5)P_2_, ubiquitination, and SQSTM1/p62 (Table 1) [102]. This process appears to be essential for M1 polarization of macrophages, which is characterized by metabolic reprogramming into glycolysis via mitochondrial turnover as well.

### l-MI-recycling endosome-phagy (l-MI-REphagy)

Our body is constantly exposed to pathogens and equipped with a highly elaborate immune system to fight them [103]. The first line of defense is the innate immune system, which has evolved to detect conserved microbial molecular patterns, generally dubbed pathogen-associated molecular patterns (PAMPs), through pattern recognition receptors (PRRs). The binding of PRRs to PAMPs activates intracellular signaling cascades that lead to the expression of proinflammatory cytokines, type I interferons, and other antiviral proteins that coordinately eliminate pathogens and infected cells.

Cytosolic double-stranded DNA (dsDNA) is one of the PAMPs [75], which can have microbial origin or leaked out from the nucleus and/or mitochondria. cGAS (cyclic GMP-AMP synthase) [104] is a PRR that upon binding dsDNA synthesizes cyclic GMP-AMP (cGAMP) from GTP and ATP [105]. STING1 (stimulator of interferon response cGAMP interactor 1) is an ER-localized transmembrane protein [106] that binds cGAMP and activates TBK1 (TANK binding kinase 1) [107], which in turn phosphorylates the transcription factor IRF3 (interferon regulatory factor 3). IRF3 stimulates the transcription and expression of type I interferons [108]. STING1 also induces a proinflammatory response via NFKB/NF-kB (nuclear factor kappa B) via the activation of TBK1 and IKBKE/IKKε (inhibitor of nuclear factor kappa B kinase subunit epsilon) [109,110]. After the binding to cGAMP, STING1 exits the ER and traffics to the Golgi, recycling endosomes (REs), and lysosomes [111–114]. It has been shown that STING1 activates the TBK1-IRF3 signaling axis at the trans-Golgi network (TGN) [113,115,116] and that STING1 is subsequently degraded in lysosomes [112]. Airyscan super-resolution microscopy and CLEM suggested that STING1-positive vesicles with an RE origin are directly encapsulated into LAMP1-positive compartments. Since STING1 colocalization with lysobisphosphatidic acid, a phospholipid enriched in LEs or with EEA1 (early endosome antigen 1)-positive vesicles does not significantly change during STING1 trafficking to lysosomes, this observation infers that LEs do not mediate STING1 encapsulation and degradation. Knockdown of TSG101 or VPS4 results in the accumulation of STING1 vesicles in the cytosol, leading to a sustained type I interferon response. STING1 is subjected to K63-linked ubiquitination at lysine 288 at the REs, and this ubiquitination is required for STING1 degradation. Importantly, STING1 degradation does not require *ATG* genes [112,117,118]. These results illustrate the critical role of l-MI-autophagy of REs (MI-REphagy) in preventing hyperactivation of innate immune signaling [8]. The involvement of the ESCRT system in STING1 degradation has also been confirmed by other recent studies (Table 1) [119,120].

Several mutations in ESCRT complex, including VPS37A in ESCRT-I [121], CHMP1A [122] and CHMP2B in ESCRT-III [123,124], VPS4A [125,126], and the ESCRT-associated protein UBAP1 (ubiquitin associated protein 1) [127] have been reported to cause neurodegenerative diseases. Interestingly, a recent study shows that the expression of the disease-associated UBAP1 variant leads to both disruption of basal STING1 degradation and increased STING1-dependent inflammation [120]. Thus, the impaired MI-REphagy that targets STING1 may contribute, at least in part, to the pathogenesis of neurogenerative diseases caused by mutations of the ESCRT components.

### l-MI-lysophagy

The term MI-lysophagy refers to a selective lysosomal membrane turnover by MI-autophagy. Recent studies have revealed that the lysosomal membrane turnover is induced by either glucose starvation or lysosomal damage in mammalian cells [128]. Intriguingly, this selective turnover of lysosomal membrane requires non-canonical function of lipidated MAP1LC3/LC3 (microtubule associated protein 1 light chain 3) proteins on lysosomal membranes, which promotes the formation of intralumenal vesicles (ILVs) insides the lysosomes and is essential to regulate lysosome size and function [128]. In contrast, some lysosomal proteins such as RNF152 (ring finger protein 152) and LAPTM4A (lysosomal protein transmembrane 4 alpha) are internalized and degraded in lysosomes at steady state condition in ESCRT-dependent but LC3 lipidation independent manner (Table 1) [129]. In parallel, other works have shown that the function of ESCRT complexes is critical to repair damaged lysosomes and this repair precedes macrolysophagy, which sequesters whole damaged lysosomes within autophagosomes [130,131]. Recent data demonstrated that the molecular mechanism governing l-MI-lysophagy and the ESCRT-dependent repair during lysosomal damage are overlapping [30]. Among mammalian Atg8-protein family members (ATG8s), lipidation of proteins belonging to the GABARAP (GABA type A receptor-associated protein) subfamily are critical for the assembly of ESCRT complexes onto damaged lysosomes via an interaction with PDCD6IP/ALIX/programmed cell death 6 interacting protein) [30]. In addition, STK38 (serine/threonine kinase 38), one of the AGC kinases, terminates l-MI-lysophagy by recruiting VPS4 proteins, which catalyze the disassembly of the ESCRT complexes. Knockdown of the GABARAP proteins or STK38 accelerates cellular senescence of mammalian cells and curtails lifespan in *C. elegans*, suggesting the importance of lysosomal homeostasis maintenance by l-MI-lysophagy to prevent cellular senescence and aging [30]. As mentioned above, several yeast vacuolar membrane proteins such as Ypq1, Vph1 and Pho8 are degraded by v-MI-vacuolophagy in ESCRT-dependent manner [19,73,76] and the degradation of some of these proteins also requires Atg8, suggesting that similar mechanisms are partly shared within eukaryotes. Further studies are required to understand differences and similarities in l-MI-lysophagy induction mechanisms under different conditions, as well as their physiological significance.

## Endosomal microautophagy (e-MI)

### Origins of mammalian e-MI

The term e-MI was coined in 2011 to describe the entrapment of cytosolic proteins in invaginations forming in the surface of the LEs/MVBs in mammalian cells, thus following a type 2 MI mechanism [24]. Early studies using metabolic labeling in organs such as the liver established MI-autophagy as a constitutively active process of protein degradation, in clear contrast to macroautophagy, which is predominantly activated during nutrient deprivation [2,3]. In vitro studies using an endolysosomal fraction isolated from rat liver reconstituted what was described as micropinocytosis and degradation of exogenously added proteins, such as ferritin [132].

As described in previous sections, studies in yeast identified a similar sequestration of cytosolic cargo directly by the vacuole, i.e., v-MI, leading to the identification of molecular effectors, some unique to this process and others shared with macroautophagy. However, the molecular dissection of e-MI in mammals lagged behind. One factor contributing to this delay was the gradual refinement in the characterization of the endocytic/lysosomal system, which brought attention to endosomal functions unrelated to autophagy. For example, LEs/MVBs became tightly associated with protein secretion, particularly as key sites of exosome biogenesis [133]. Additionally, research on protein degradation in this compartment predominantly focused on ubiquitin-dependent degradation of plasma membrane proteins internalized by endocytosis [134]. The development of methods to purify secondary lysosomes from LEs/MVBs enabled the isolation of different lysosomal subtypes with distinct functions in autophagy. Some secondary lysosomes were found to be highly efficient in autophagosome-lysosome fusion [135], whereas another subset was shown to translocate proteins directly across the lysosomal membrane via CMA [136].

The morphological resemblance of LEs/MVBs to the original descriptions of MI-autophagy in mammals sparked interest in investigating whether autophagic mechanisms also operate in this compartment. Blocking macroautophagy initiation, e.g., through ATG7 or ATG5 knockdown, effectively reduced the presence of a subset of cytosolic proteins detected in the lumen of LEs/MVBs, which had reached this compartment via direct fusion with autophagosomes to form amphisomes [24]. However, other cytosolic proteins were still detected in the lumen of LEs/MVBs even when both macroautophagy and CMA were inhibited. Using a combination of biochemical and imaging-based approaches, it was uncovered that these cytosolic proteins were internalized into LEs/MVBs through a MI-autophagy-like process, which was subsequently termed endosomal microautophagy (e-MI) [24].

### Types of mammalian e-MI

The initial study on e-MI identified the coexistence of both a nonselective and a selective form of e-MI in mammalian cells. In the nonselective form, soluble cytosolic proteins were sequestered in bulk into the forming vesicles, whereas in the selective form, the chaperone HSPA8/HSC70, a constitutive member of the HSP70 protein family, delivered only proteins containing a pentapeptide identical to one of CMA [137]. The resemblance between e-MI and the vesiculation occurring in LEs/MVBs for exosome biogenesis led to an investigation of the possible involvement of the ESCRT system in both processes. Blocking the ESCRT-I component TSG101, along with the ESCRT-associated proteins VPS4 and PDCD6IP/ALIX, completely halted e-MI, indicating that this pathway shares some molecular components with the MVBs and exosome biogenesis [24].

Shortly after the description of HSPA8/HSC70-dependent mammalian e-MI (thereafter referred as HSPA8/HSC70-dependent e-MI), studies using quantitative proteomics to analyze the fraction of the proteome degraded upon starvation led to the identification of starvation-induced e-MI. This process appears to be responsible for the rapid, selective degradation of SARs during the first 30 min of nutrient deprivation and depends on a subset of ESCRT complexes as well [25]. Further analysis of SARs revealed a form of e-MI mediated by TOLLIP, in which this SAR and its associated cargo are degraded in LEs/MVBs [138]. Like other variants of mammalian e-MI, this process also appears to be independent of the core ATG machinery.

In mammals, the degradation of ferritin particles, necessary for the release of iron bound to this carrier protein, has also been shown to occur, at least in part, through e-MI. In this process termed MI-ferritinophagy [139], ferritin particles are first organized into liquid-like condensates with the assistance of NCOA4 (nuclear receptor coactivator 4) before being internalized into either autophagosomes (macroferritinophagy) or endosomes (e-MI) in a TAX1BP1 (Tax1 binding protein 1)-dependent process [139]. This latter process also requires a subset of ATG proteins, namely RB1CC1/FIP200, ATG9A and PIK3C3/VPS34 [140].

Other forms of cytosolic protein internalization into LEs/MVBs have been described. For example, under conditions of iron depletion, GAPDH (glyceraldehyde-3-phosphate dehydrogenase) is internalized into early endosomes (EEs) in an ESCRT (TSG101 or VPS4)- and 70-dependent manner for trafficking outside the cell [141]. However, in the following sections and Table 2, we will focus on cargo internalization into endocytic compartments that leads to degradation, as this is a requirement for classification as an autophagic pathway.

### HSC70-dependent mammalian e-MI

**Figure 3.**
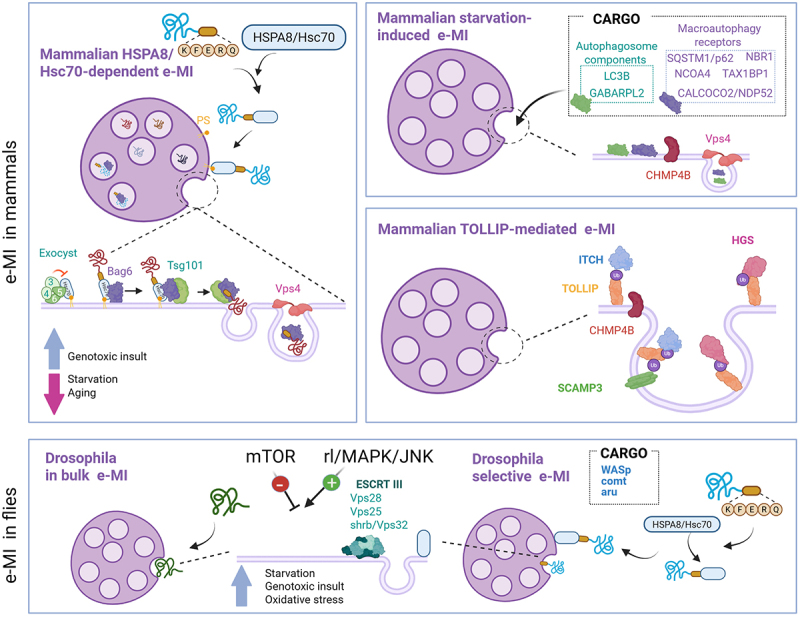
Different types of e-MI in mammals and flies. Schemes depict known molecular components, regulators and cargo for each of these processes.

Substrate targeting by HSPA8/HSC70 in a KFERQ-dependent manner is the defining characteristic that distinguishes this type of e-MI from other mammalian e-MI processes (Table 2). While the KFERQ-like motif is necessary and sufficient for substrate targeting via CMA, it has been shown to be necessary but not sufficient in the case of e-MI [24]. Furthermore, instead of the docking of the HSPA8/HSC70-cargo complex on LAMP2A, as described for CMA, in e-MI, HSPA8/HSC70 binds to phosphatidylserine (PS) on the LE membrane through electrostatic interactions and consequently is LAMP2A-independent [24] (Figure 3). Follow-up studies identified the C terminus of the HSPA8/HSC70 LID domain as the structural interface responsible for interacting with endosomal PS [142]. The vesicle-mediated mechanism of cargo internalization makes substrate unfolding, which is required for CMA, unnecessary for HSPA8/HSC70-dependent e-MI [24].

The current understanding of HSPA8/HSC70-dependent e-MI has been facilitated by the ability to reconstitute this process *in vitro* using isolated LEs/MVBs, as well as the development of a fluorescent reporter system incorporating KFERQ motifs and the split-VENUS methodology, which allows for monitoring of e-MI in cultured cells [143]. Beyond HSPA8/HSC70, recent studies have identified the involvement of a second chaperone, BAG6 (BAG cochaperone 6), in HSPA8/HSC70-dependent e-MI. BAG6 associates with the LE membrane and is required for substrate internalization (Figure 3). In fact, this chaperone binds both substrates and the ESCRT component TSG101, which determines substrate loading into the forming vesicles. Unlike HSPA8/HSC70, which is not internalized with its substrates, a fraction of BAG6 is incorporated within the vesicles and undergoes degradation along with the cargo [143]. Comparative proteomic analyses of e-MI cargo in the presence or absence of BAG6 suggest that it may not be required for all e-M substrates. HSPA8/HSC70-dependent e-MI substrates displaying BAG6 dependence include proteins involved in RNA transport, translation, and processing in the ER [144].

Components of exocyst complexes I and II inhibit HSPA8/HSC70-dependent e-MI directly at the LE membrane, where they interact with HSPA8/HSC70 and BAG6 [145]. The current working model proposes that exocyst components directly sequester a portion of LE/MVB-associated HSPA8/HSC70, thereby limiting its contribution to e-MI (Figure 3). The inhibitory function of the exocyst in HSPA8/HSC70-dependent e-MI appears to be closely related to its physiological role in the tethering of secretory vesicles to the plasma membrane for exocytosis. This connection explains why, when the degradation of e-MI cargo in LEs/MVBs is not possible, the undegraded cargo is instead secreted extracellularly.

Some of the earliest identified substrates for HSPA8/HSC70-dependent e-MI (e.g., GAPDH, RNase A, MAPT/TAU (microtubule associated protein tau), PTP4A2/PRL2 (protein tyrosine phosphatase 4A2) have also been reported to undergo degradation in lysosomes via CMA [145,146]. In fact, comparative proteomic analyses of lysosomes and LEs/MVBs demonstrated that both pathways can degrade overlapping portions of the cellular proteome, albeit with different efficiencies [145]. Interestingly, these analyses have revealed a preferential degradation by e-MI of components of other proteolytic systems, including the proteasome and mitophagy, ribosome-related proteins, and proteins involved in carbohydrate metabolism [145]. For some newly identified substrates, such as ULK2, e-MI degradation depends on NEDD4L (NEDD4 like E3 ubiquitin protein ligase)-mediated ubiquitination [147]. However, whether this or other post-translational modifications are required for all or only a subset of e-MI substrates remains to be determined.

Recent studies support a possible role of e-MI in protein quality control. In addition to the potential BAG6-dependent role of e-MI in the quality control of proteins processed in the ER, certain aggregation-prone proteins, such as mutant huntingtin, have been shown to undergo degradation through e-MI in mammals before forming aggregates [148]. This degradation is independent of LAMP2A or ATG7 but depends on ESCRT components in complex 0 [STAM (signal transducing adaptor molecule) and STAM2], I (TSG101), III (VPS4A and PDCD6IP/ALIX), and RAB35 [148]. Dependence on HSPA8/HSC70 has not been established in this context, despite huntingtin containing multiple KFERQ-like motifs in its sequence.

Although further studies are required to elucidate the physiological relevance of e-MI in mammals, recent research supports the original idea that MI-autophagy is constitutively active. In fact, conditions such as starvation, which typically upregulate other types of autophagy to their maximal levels, lead to reduction in e-MI activity [144]. Interestingly, BAG6 may play a crucial role in the inverse regulation of e-MI and CMA in response to starvation (Figure 3). Starvation promotes the degradation of BAG6 through CMA, likely limiting substrate internalization by e-MI under these conditions [144]. The opposing responses of e-MI and CMA to starvation suggest that e-MI plays a more active role in maintaining and regulating the cellular proteome under basal conditions while contributing less to the cellular energy requirements during nutrient deprivation. The inhibition of HSPA8/HSC70-dependent e-MI by starvation further distinguishes this process from other types of e-MI [25,138,149,150]. While e-MI does not appear to be upregulated by stressors such as oxidative stress, specific substrates such as PTP4A2/PRL2 important for bone formation, are preferentially degraded by e-MI in response to oxidative stress [146].

HSC70-dependent e-MI malfunctions with age [145]. Studies conducted both *in vivo* and *in vitro* using LEs/MVBs from rodents of different ages have revealed changes in the fraction and a reduction in the degradation rate of the proteome delivered to these organelles in older mice. Comparative proteomic analyses of LEs/MVBs from young and old mice indicate not only quantitative but also qualitative differences in the array of proteins degraded in these compartments. For example, while HSPA8/HSC70-dependent e-MI in young animals preferentially targets proteins involved in carbohydrate metabolism, aging leads to a noticeable shift toward the turnover of proteins involved in lipid metabolism [145]. Whether these changes in HSPA8/HSC70-dependent e-MI cargo result from age-related metabolic shifts or the failure of other proteolytic pathways, such as CMA, requires further investigation. Age-related turnover failure of e-MI cargo is associated with the aberrant internalization of HSPA8/HSC70 along with the cargo inside MVBs. Age-dependent glycation of HSPA8/HSC70 at the surface of LEs/MVBs appears to underlie these altered dynamics, increasing HSPA8/HSC70’s interaction with substrates and components of the exocyst complex in these compartments [145]. The reduced degradation of e-MI-internalized cargo in LEs with age correlates with an increased extracellular release of undegraded cargo [145]. Indeed, experimental inhibition of this turnover is sufficient to trigger increased secretion. The enhanced association of RALA (RAS like proto-oncogene A), an additional exocyst complex component, with the LE/MVB membrane in aging promotes the docking of these compartments with the plasma membrane and subsequent cargo release in exosomes, effectively switching LEs/MVBs from degradative to secretory compartments [145]. Notably, under physiological conditions in young organisms, selective degradation of RALA by CMA prevents this shift toward secretion.

This transition of LEs/MVBs from degradative e-MI compartments to secretory organelles has also been observed in pathological contexts associated with proteotoxicity, such as tauopathies. Although MAPT/TAU is an optimal substrate for both CMA and HSPA8/HSC70-dependent e-MI, pathogenic MAPT/TAU variants inhibit these pathways by blocking the internalization and degradation of other substrates [151]. Experimental CMA blockage, as the one described in tauopathies, leads to the re-routing of pathogenic MAPT/TAU variants to LEs/MVBs in an HSPA8/HSC70- and ESCRT-dependent manner. However, their failure to undergo degradation in these compartments results in extracellular release, contributing to disease propagation [152]. Interestingly, upon CMA blockage, only a subset of its substrates is re-routed to HSPA8/HSC70-dependent e-MI, despite all of them containing a KFERQ-like motif [145]. Comparative proteomic studies suggest that some CMA functions, such as its recently proposed role in the regulation of protein translation and folding [153] as well as a previously unrecognized role in ER protein localization and processing, may partially be compensated by HSPA8/HSC70-dependent e-MI when CMA is impaired [144]. These findings support the notion that HSPA8/HSC70-dependent e-MI and CMA are non-redundant pathways that can only partially compensate for each other.

### Starvation-induced mammalian e-MI

Contrary to the constitutive nature of HSPA8/HSC70-dependent e-MI, the ability to be maximally upregulated in response to starvation is the main characteristic of a different type of mammalian e-MI, termed starvation-induced e-MI (Table 2). The identification of this pathway in transformed and non-transformed mammalian cell lines originated from the interest in determining whether nutrient deprivation-induced macroautophagy was a selective or bulk process. Focusing on the acute phase of amino acid starvation allowed the identification of a subset of cytosolic proteins that undergo rapid degradation in LEs/MVBs. These proteins included SARs such as SQSTM1/p62, NBR1, TAX1BP1, CALCOCO2/NDP52 and NCOA4, and members of the ATG8 protein family like LC3B and GABARAPL2, which are required for cargo selection and autophagosome biogenesis [25]. Extensive knockdown studies enabled a detailed molecular characterization of this type of e-MI [25], revealing that it depends on the regulatory ATPase VPS4A and some ESCRT-III components (CHMP4B) but not others (CHMP3/VPS24, CHMP4A or CHMPAC) (Table 1). However, this type of e-MI does not require ESCRT-0 [HGS/HRS (hepatocyte growth factor-regulated tyrosine kinase substrate)], ESCRT-I (TSG101 or VPS28), or ESCRT-II (VPS22/EAP30). Knockdown of HSPA8/HSC70 also confirmed the independence of this type of e-MI from this chaperone [25].

Interestingly, the dependence on macroautophagy components for starvation-induced e-MI appears to be cargo-dependent. For instance, degradation of SQSTM1/p62 and CALCOCO2/NDP52 by this pathway requires functional ATG7, ATG5, and lipidated ATG8 proteins, whereas substrates like NBR1, TAX1BP1, and NCOA4A undergo degradation even in the absence of these ATG components. While the targeting mechanisms for this type of e-MI are not yet elucidated, the detection of SQSTM1/p62 at the membrane of LEs, even when MVB formation was blocked, suggests a high affinity of these proteins for membranes. This affinity could explain their rapid degradation within the first minutes of amino acid deprivation. In the case of SQSTM1/p62, the dependence on an intact LIR suggests that members of the ATG8 protein family may be involved in substrate targeting in starvation-induced e-MI.

Both starvation-induced e-MI and macroautophagy are upregulated in the absence of nutrients, but their kinetics are quite different. Starvation-induced e-MI follows degradation kinetics like those of membrane proteins, peaking earlier than macroautophagy and being completed within three h [25]. Additionally, unlike macroautophagy, starvation-induced e-MI does not require inactivation of MTOR (mechanistic target of rapamycin kinase) complex 1 (MTORC1), suggesting that changes in extracellular amino acid levels, rather than intracellular pools, serve as the trigger for this type of e-MI.

Although the physiological relevance of starvation-induced e-MI is not yet fully understood, its distinctive preference for degrading SARs has led to the proposal that this pathway could serve as a mechanism to prevent the activation of selective macroautophagy during nutrient deprivation, shifting cellular resources toward bulk macroautophagy under these conditions [25].

### TOLLIP-dependent mammalian e-MI

A recent study aiming to identify the autophagic degradome targeted by SARs that contain ubiquitin-binding domains led to the identification of a form of e-MI in mammals that depends on the SAR TOLLIP [138]. Opposite to the other SARs examined in this study, the authors noted that TOLLIP-containing vesicles were highly enriched in endosomal marker proteins. Cells knocked out for TOLLIP failed to degrade specific cargo proteins such as HGS/HRS, SCAMP3 (secretory carrier membrane protein 3) and ITCH (itchy E3 ubiquitin protein ligase), all of which interact with TOLLIP through its ubiquitin binding domain. Immunofluorescence studies showed the presence of TOLLIP in LEs/MVBs and the selective engulfment of TOLLIP cargo within these compartments. Importantly, both findings were confirmed biochemically through protease protection assays. TOLLIP-dependent e-MI is insensitive to macroautophagy inhibition but depends on ESCRT components such as CHMP4B [138]. TOLLIP-dependent e-MI is defective when MVB integrity is compromised through treatment with the compounds U18666A or GW4869 [138]. To what extent TOLLIP-dependent delivery of cargo to LEs/MVBs only occurs in fed cells and is abolished during starvation as observed in HSPA8/HSC70-dependent e-MI, remains to be addressed.

### e-MI-aggrephagy

While there is not an undisputable proof that protein aggregates can be delivered in the degradative interior of the compartments of the endolysosomal system by MI-autophagy, some compelling evidence suggests the existence of e-MI-aggrephagy pathways (Table 2). The ability of e-MI to degrade proteins even when organized into oligomers [24] has inspired studies aiming to target amyloid oligomers for clearance via e-MI *in vivo*. This approach involves feeding experimental animal models with peptides containing KFERQ-like sequences primed for HSPA8/HSC70 recognition [154]. Co-aggregation of these peptides with pathogenic oligomers facilitates their recognition by HSPA8/HSC70 and subsequent targeting to e-MI. A similar process may take place physiologically, as a recent study has shown that amyloid-like aggregates formed by a truncated version of MAPT/TAU carrying the frontotemporal dementia-associate P301L mutation that enhances aggregation propensity, are partially degraded by e-MI [155]. This process occurs at EEA1-positive EEs, requires the ESCRT system but does not involve ATG proteins, and it is impaired by an autosomal dominant hereditary spastic paraplegia (HSP)-associated mutation in the ESCRT-I subunit UBAP1. MAPT/TAU pathology has been reported in patients with HSP. Cargo recognition requires ubiquitylation of the repeat domain of MAPT/TAU, which is then recognized by the ESCRT protein TGS101, proposed as a SAR for this type of e-MI-aggrephagy [155]. Importantly, this study also showed that aggregates formed by Parkinson disease-associated SNCA (synuclein alpha) carrying the A53T mutation (SNCA^A53T^) but not by a polyglutamine stretch of 97 units that is associated with Huntington disease, strongly accumulate in absence of ESCRT system components [155].

Differences other than the type of protein that becomes aggregate, may also be determinants on potential clearance of aggregates by e-MI-aggrephagy. Thus, for example, overexpressed SNCA was found to be mostly transported in the lumen of RAB5-positive EEs in a ubiquitin-, NBR1- and ESCRT-dependent manner in cell lines and neurons [156]. However, formation of seeded aggregates resulted in reduced colocalization of the ubiquitinated SNCA in endosomes and redistributed toward inclusions [157]. It is possible that intrinsic characteristics of different types of aggregates play a role in their potential of undergoing e-MI-aggrephagy.

Altogether, these studies suggest the existences of e-MI-aggrephagy processes that require ESCRT complexes, but not core ATG proteins. The involvement of HSPA8/HSC70 and whether TSG101 is a universal receptor for all types of e-MI-aggrephagy still remains to be established.

### e-MI-ERphagy

As described above, in yeasts, the ER can be degraded by v-MI-ERphagy. Morphometric studies of hepatocytes from rats, rabbits and dogs acutely exposed to compounds such as the antiepileptic drug phenobarbital, revealed that the stress-induced ER swelling is reversed upon interruption of the pharmacological treatment and it is accompanied by the lysosomal clearance of excess ER to reestablish steady-state ER size and functions [158]. The mechanistic characterization of ERphagy had to await over 40 years for the identification of the selective ERphagy receptors, i.e., the ER membrane-associated proteins that promote fragmentation and, upon engagement of cytoplasmic ATG proteins, delivery of the ER portions to degradative endolysosomal compartments exposing RAB7 and LAMP1 (lysosomal associated membrane protein 1) at the limiting membrane[5]. ERphagy pathways can be triggered by *pleiotropic* signals such as nutrient restriction. These signals simultaneously activate the majority, if not all, selective autophagy receptors (SARs) that are engaged in macroautophagy pathways. However, ERphagy can also be induced by more specific cues, i.e., ER-*centric* signals, such as the lumenal accumulation of misfolded proteins [159–162] or recovery upon acute ER stresses [29,163–166]. In contrast to the *pleiotropic* cues, the *ER-centric* signals activate individual, rather than multiple ERphagy receptors [e.g., RETREG1/FAM134B (reticulophagy regulator 1), RTN3L (reticulon 3 L), or CCPG1 (cell cycle progression 1)] eliciting macro-ERphagy, but also LC3-dependent (vesicular) transport and MI-ERphagy pathways [5].

**Figure 4.**
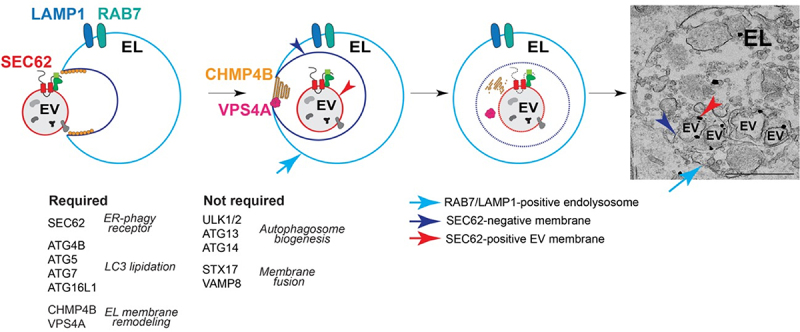
Schematic representation of piecemeal MI-ERphagy, modified from[170]. Gene products required or dispensable for Recov-ERphagy are listed. Arrows and arrowheads in the schematics and in the immunogold-labelled TEM preparation show the membrane of the RAB7 and LAMP1-positive endolysosome (EL, light blue), of the endolysosomal invagination (dark blue), and of the ER vesicle (EV, red). Gold particles label endogenous SEC62.

In mammalian cells, treatment with cyclopiazonic acid (CPA), a reversible inhibitor of the ER calcium pumps ATP2A/SERCA (ATPase sarcoplasmic/endoplasmic reticulum Ca2+ transporting) [167], causes expansion of both the ER and the distance between the inner and the outer nuclear membrane [163,164,168]. Upon removal of CPA the ER returns to pre-stress morphology and protein content [163], through a program that is also characterized by the delivery of ER portions within RAB7- and LAMP1-positive endolysosomal compartments (Table 2). Correlative light electron microscopy (CLEM) and transmission electron microscopy (TEM) analyses have revealed that the endolysosomal compartments engulf ER-derived vesicles through MI-ERphagy (Figure 4) [163,164,168,169]. This process, which has been named Recov-ERphagy, depends on ATG proteins belonging to the ATG12 conjugation and ATG8 protein lipidation machineries such as ATG4B, ATG5, ATG7 and ATG16L1, but not other proteins essential for macroautophagy like ULK1 (unc-51 like autophagy activating kinase 1), ULK2, ATG13 and ATG14 as well as SNARE proteins that regulate autophagosome fusion, including STX17 (syntaxin 17) and VAMP8 (vesicle associated membrane protein 8) (Figure 4) [29]. Consistent with the type 2 inward budding of MI-ERphagy, Recov-ERphagy also requires the ESCRT-III components CHMP4B (charged multivesicular body protein 4B) and VPS4 [29]. The presence of ESCRT proteins in the DO compartments of MI-ERphagy makes likely that this process takes place in LE/MVB, and hence its inclusion as a type of e-MI. However, future studies are required to determine if some forms of MI-ERphagy deliver the ER cargo directly to lysosomes, leaving open the possibility of l-MI-ERphagy. Interestingly, Recov-ERphagy requires the SEC62 (SEC62 preprotein translocation factor) SAR and its conserved LC3-interacting region (LIR), but no other ERphagy SARs such as RETREG1/FAM134B [163,164,168]. However, it remains unclear which is the function of the LC3 proteins in Recov-ERphagy and whether LC3 proteins are lipidated on either the ER-derived fragments or the target endolysosomal compartments.

It has been shown that the misfolded mutant form of COL1A2 (collagen type I alpha 2 chain), which carries a glycine to cysteine substitution at position 610 and has been associated osteogenesis imperfecta [171], is segregated in LC3- and SQSTM1/p62 (sequestosome 1)-positive ER exit sites (ERES). CLEM analyses have revealed that these sites containing misfolded COL1A2^G610C^ are directly engulfed by endolysosomes in a still mechanistically poorly defined catabolic pathway that has been named ERES MI-autophagy [172]. It remains unclear which ATG proteins and ERphagy SARs are involved in ERES MI-autophagy and if the ESCRT machinery is required for this process.

Recent evidence has revealed that in infected cells, the human murine leukemia virus (MLV) glycoGag protein triggers the clearance of SERINC5 (serine incorporator 5), a host cell restriction factor that integrates into the viral envelope at the plasma membrane inhibiting viral cell entry. The proposed mechanism consists in the enhancement of RETREG1/FAM134B-driven MI-ERphagy resulting in SERINC5 turnover that decreases its cell surface expression. Surprisingly, all the examined *ATG* genes, including *PIK3C3 (*phosphatidylinositol 3-kinase catalytic subunit type 3*)/VPS34*, *BECN1* (beclin 1) and members of the two ubiquitin-like conjugations systems such as *ATG3*, *ATG5* and *ATG7*, are dispensable [173]. It remains unknown whether this process requires the ESCRT machinery.

### e-MI in flies

The existence of an alternative form of autophagy in *Drosophila* was unknown, until not long-ago when evidence for constitutive and stress-induced e-MI became available [27,28]. Studying the membrane deformation function of Hsc70-4, one of the fly paralogs of HSPA8/HSC70, evidence for KFERQ-dependent, constitutive e-MI in the larval neuromuscular junction was found (Table 2) [27]. Manipulation of Hsc70-4 activity by mutation and overexpression altered the steady state levels of several KFERQ motif-containing proteins including the Wiscott Aldrich Syndrome protein WASp and comt (comatose), the fly homologue of NSF (N-ethylmaleimide sensitive factor), but not of several proteins lacking the KFERQ sequence. This effect of Hsc70-4 requires its membrane deformation activity, but not its chaperone function, consistent with Hsc70 being antagonized by the co-chaperone Sgt (small glutamine rich tetratricopeptide containing protein), which promotes Hsc70-4 chaperone function. Physiologically, the e-MI function of Hsc70-4 promotes the faster turnover of comt and likely other synaptic proteins in a KFERQ-dependent manner, leading to a larger pool of neurotransmitter vesicles ready for release [27].

In parallel, Mukherjee *et al*. described starvation induced e-MI in the larval fat body, which functions similar to the mammalian liver and adipose tissue [28]. Using a KFERQ-sensor originally developed for the study of CMA in mammals [174], it was shown that in the fat body, e-MI depends on Hsc70-4, and components of the ESCRT machinery, including Vps28, Vps25, and shrb/Vps32, suggesting that ESCRT complexes I to IIII are essential. In addition to a portion of e-MI that is KFERQ-dependent, there is a fraction of cargo that is sequestered in bulk [28,175]. Like CMA in mammals, this e-MI is induced by prolonged exposure to stress. Refeeding experiments showed that, in contrast to macroautophagy that peaks between 1 and 4 h of starvation [176], e-MI induction requires at least 12 h of starvation, which led to the suggestion that *Drosophila* e-MI shares functions that in mammals are fulfilled by both e-MI and CMA [28]. Importantly, Lamp1, the bona fide homolog of mammalian LAMP1/LAMP2 is dispensable for e-MI, showing that flies have no CMA [177].

Interestingly, in addition to the negative regulation of e-MI by TOR, stress and genotoxic stress also induced e-MI in the fat body, while ER stress did not, suggesting specificity for this type of stress eliciting e-MI [175]. Mechanistically, genetic experiments showed that reactive oxygen species (ROS)-induced e-MI is mediated by rl/MAPK/JNK (rolled) signaling. The regulation of e-MI by DNA damage is less clear. While mutations in *tefu/ATM* (telomere fusion), *mei-41/ATR* (meiotic 41), *grp*/*CHEK1* (grapes) and *lok/CHEK2* (loki) each increase e-MI in response to the accumulation of DNA damage, none of these DNA damage response kinases is uniquely required for e-MI induction, suggesting potential redundancy or a different mechanism [175].

Additional evidence for a basal e-MI was provided by the finding that Aru (Arouser), a member of the EPS8 (EGFR pathway substrate 8, signaling adaptor) protein family, is degraded in lysosomes of fed larvae in a manner dependent on Hsc70-4, the ESCRT machinery and Atg1 and Atg13. Importantly, Aru degradation is independent of Atg8a and Atg7, strongly suggesting it is an e-MI substrate *in vivo* [178]. Indeed, mutating its KFERQ motif stabilized Aru under fed conditions. Unexpectedly, starvation or mTor inhibition stabilizes Aru and allows this protein to regulate lipid metabolism, leading to an increased resistance to starvation [178]. It will be interesting to determine in future studies which proteins are subject to basal versus stress induced e-MI, and how some, such as aru, are protected from degradation by the latter.

Recent studies in *Drosophila* have described the ability of an additional chaperone, Csp/DNAJC5 (Cysteine string protein), to mediate the delivery of misfolded proteins to endolysosomal compartments in an ESCRT-dependent manner [179]. This process was also detectable in mammalian cells in culture and, as it is the case for BAG6 in mammalian HSPA8/HSC70-dependent e-MI, Csp/DNACJ5 was also internalized and degraded with the cargo. However, since cargo targeting/internalization is still preserved when using a DNAJC5 variant unable to bind HSPA8/HSC70, it is likely that this type of e-MI is different from HSPA8/HSC70-dependent e-MI [179]. Whether this process overlaps with bulk e-MI, as described both in mammals and flies, requires further investigation.

## Cross-talk between MI-autophagy and other autophagic processes

In recent years, the cross-talk between MI-autophagy and macroautophagy have been increasingly appreciated, from their respective molecular mechanisms to biological functions in various stress conditions and in the model organisms, from yeast to mammalian cells [8,91]. Despite the efforts, there are some important questions on the relationship between these two forms of autophagy that remain to be addressed. For instance, what is the nature of correlation between MI-autophagy and macroautophagy? Do they co-exist in the same model under the same context? How do the cells select between these two forms? How are they functionally coordinated? How can they be targeted differently or commonly in various disease models for the purpose prevention and therapy?

A similar cross-talk has been identified between HSPA8/HSC70-dependent e-MI and CMA, showing partial compensation for each other [144,145]. Experimental blockade of e-MI in cultured cells results in CMA upregulation, and inhibition of CMA, both *in vitro* and *in vivo*, in mouse models, leads to activation of HSPA8/HSC70-dependent e-MI. However, these two pathways are not redundant since, as described in the previous sections, upon CMA disruption, only a subset of proteins degraded by this pathway are rerouted to e-MI [144]. At the molecular level, cross-talk may be facilitated by the fact that CMA and HSPA8/HSC70-dependent e-MI share the same targeting motif and binding chaperone. Furthermore, BAG6, which is required for HSPA8/HSC70-dependent e-MI of a subset of the proteome, is degraded by CMA, thus limiting the amount of this co-chaperone available for e-MI under conditions such as starvation [144]. Additional molecular components modulating this crosstalk and the cellular conditions that promote coordinated changes in CMA and e-MI activities require future investigation.

### Different engagement of ATG proteins

As discussed in the earlier sections, it is generally believed that these two forms of autophagy differently engage some of the key autophagy regulators such as the ATG proteins. On the one hand, v-MI-autophagy induced by nutrient starvation or MTORC1 inactivation triggers the Nem1/Spo7-Pah1 axis, without the participation of other key macroautophagy players, including Atg1, Atg7 or Atg8, in budding yeast [180]. On the other hand, yeast studies have shown that some forms of MI-autophagy also require the participation of Atg proteins [6]. For example, an earlier study by Krick and coworkers found that v-MI-nucleophagy involves the core *ATG* genes, including *ATG7*, *ATG8*, and *ATG9* [22].

One possible explanation for such discrepancy is that Atg proteins may subject to different modifications or work with different partners to form different complexes. For example, ATG7 undergoes deacetylation in response to many different stimuli in mammals, a process enhancing its affinity to ATG3 and promoting ATG8 protein lipidation, leading to both canonical macroautophagy and LC3-associated MI-autophagy, mainly for the selective turnover of lysosome membrane proteins to maintain lysosome activity [181].

Another possibility is that the same ATG proteins act at different subcellular location or are association with different membranes or organelles, leading to different forms of autophagy. One example is that, under glucose starvation or osmotic stress of lysosomes, lipidation of LC3 proteins onto lysosomal membranes trigger l-MI-autophagy with a concomitant formation of ILVs [128,182].

### Different engagement of endosomes and lysosomes

Although it is well known that both MI-autophagy and macroautophagy need the participation of endosomes and lysosomes as the final destinations for the degradation of their cargos, these endocytic organelles are engaged in these two forms of autophagy differently. Recent studies have demonstrated the importance of regulating vacuolar/lysosomal morphology via fission and fusion in budding yeast and in *C. elegans* [183,184]. The morphological dynamics of lysosome is regulated by the nutritional status and controlled by MTORC1, as well as by the specific lysosomal scission factors like *C. elegans* HPO-27, a homolog of human MROH1 [183,185]. At present, it remains to be tested whether this link between lysosomal morphology and forms of MI-autophagy also exists in mammalian cells.

### Different cellular contexts with different types of stress

One key factor in determining the forms of autophagy is the cellular context including the cell type, the nature of the stress and the presence or absence of some key autophagy factors. At present, there is convincing evidence suggesting that the choice between MI-autophagy or macroautophagy is stimulus-specific in the same model; the different nature of stress may cause different forms of autophagy. For instance, the yeast proteasome storage granules are sent to vacuole for degradation via a v-MI-autophagic process under glucose starvation [186], while proteasomes are turned over by macroautophagy upon nitrogen deprivation [187]. Such stimulus-dependent selection of autophagy forms has been verified in yeast [12,13,188–190]. Therefore, it is thought that MI-autophagy is part of the constitutive response specific to glucose starvation, even in the presence of an intact macroautophagy machinery. Currently, the molecular mechanisms regulating the type of autophagy in a stimulus-specific manner are poorly understood, especially in mammals.

### Different kinetics

Another interconnection between MI-autophagy and macroautophagy is their different kinetics in the same cell type under the same stress conditions. There is evidence showing that these two forms of autophagy could occur at the same time in response to the same stress. For instance, macronucleophagy and v-MI-nucleophagy take place in parallel, but targeting different cargos in yeast under nitrogen starvation [52]. It appears that MI-autophagy usually occurs first and faster, while macroautophagy happens slower and at later time points upon stress [91]. As previously explained, it has been found that HSPA8/HSC70-independent MI-autophagy occurs as an early response to amino acid starvation to degrade some key SARs, as a preparation for a massive induction of bulk macroautophagy [25]. This result indicates that both MI-autophagy and macroautophagy are coordinated temporally. More evidence has recently been found in yeast: MI-ERphagy is the first response to be triggered by starvation to clear aberrant membrane proteins, before induction of macro-ERphagy to degrade bulky components [191,192]. In fact the slower nature of macroautophagy is somehow understandable, based on the fact that this pathway involves multiple steps that leads to the formation and fusion of autophagosomes, and those require a larger amount of energy and lipids [193]. Additionally, sustained macroautophagy also requires gene transcription and protein synthesis.

### Coordination for the selection of cargos by MI-autophagy and macroautophagy

In addition to the difference in the way of cargo delivery, the nature of the cargos targeted by MI-autophagy and macroautophagy is often very different as well. Multiple factors dictate which type of autophagy degrades a cargo, including its size, location and post-translational modifications. As described in a previous section, Otto and Thumm observed that macronucleophagy and MI-nucleophagy target different forms of micronuclei in nitrogen starved yeast [52]. While macronucleophagy degraded smaller micronuclei, larger parts of the nucleus was directly engulfed by vacuoles via MI-nucleophagy [52]. In mammalian cells, intracellular insoluble or aggregated MAPT/TAU can mainly be degraded via macroautophagy [194,195], while soluble neurotoxic MAPT/TAU can be cleared by MI-autophagy by both CMA and e-MI [151,196]. Furthermore, post-translational modification of MAPT/TAU by acetylation impairs its degradation by MI-autophagy and convey it to macroautophagy turnover [152]. Another important factor in determining how a specific cargo is eliminated is the presence or absence of SARs. It has been reported that SQSTM1/p62-postive cargo proteins are usually degraded by macroautophagy, whereas TOLLIP-positive cargo proteins are mainly subjected to MI-autophagic degradation [138]. Thus, both SQSTM1/p62 and TOLLIP, and possibly other SARs, may act as key regulators in determining by which types of autophagy a cargo is turned over.

### Complementary nature between macroautophagy and MI-autophagy

Macroautophagy and MI-autophagy co-exist in the same cellular and organismal systems and are interlinked at multiple levels to mediate degradation in a coordinated and complementary manner. For instance, when macroautophagy is compromised, e-MI is enhanced to degrade the accumulating dysfunctional proteins [175]. Similarly, CMA blockage by knockdown of LAMP2A or through the inhibitory effect of pathogenic forms of MAPT/TAU, increases HSPA8/HSC70-dependent e-MI of MAPT/TAU [152]. Another example is when macromitophagy is suppressed; cells adopt MDV-mediated MI-mitophagy for the turnover of part of damaged mitochondria [197]. Moreover, knockdown of Atg7 in *Drosophila* enhances e-MI, suggesting the possibility that this pathway compensates for the loss of macroautophagy [175]. In liver tissues, the inhibition of CMA does not cause imminent dysfunctional proteostasis because of the upregulation of macroautophagy [198]. Another example is that loss of PRKCI/PKCλ/ι (protein kinase C iota) stabilizes ULK2 by impairing its MI-autophagic degradation, which in turn increases macroautophagy preventing STING1 degradation by CMA to enhance interferon signaling [147]. Although mechanistic dissection of macroautophagy and MI-autophagy in the same cellular model has always been technically challenging, it is important to explore how macroautophagy, MI-autophagy and CMA cooperate or compensate from each other to maintain cellular homeostasis and survival under various stress conditions in the same context.

Taken together, plenty of evidence shows that macroautophagy and MI-autophagy are mechanistically interlinked and functionally complement each other. Thus, further understanding of the cross-talk between them will be an important topic for autophagy research in the years to come.

## MI-autophagy-related pathways in mammals and plants

While typically associated with degradation, MI-autophagy-related pathways which involve MI-autophagy-like mechanisms, also have been shown to have important non-degradative roles (Table 3). In mammals, mechanisms related to MI-autophagy have emerged as key contributors to unconventional protein secretion (UPS), particularly through pathways that involve endosomal vesicular intermediates and are referred to as Type III UPS [199]. Another striking example of MI-autophagy-related process is the biosynthesis and storage of anthocyanin pigments in plants [200].

**Table 3.** Non-Degradative MI-Related Pathways.

MI-autophagy	Membrane deformity	Core ATG proteins	ESCRT	Other specific molecule
MI-related secretory pathway (LC3-Dependent Extracellular Vesicle Loading and Secretion, LDELS)	Type2	ATG7, ATG12, ATG3	HGS, PDC6IP, CHMP4B	NSMAF, SMPD3
ATG-independent v-MI-related pathway for anthocyanin sequestration	Type 1			
ATG-independent v-MI-ERphagy-related pathway for protein storage	Type 1			

### Unconventional secretion in mammalian cells

As described in the previous sections, the initial discovery of e-MI in mammalian cells revealed that a subset of LEs/MVBs can trap cytosolic content during e-MI for their degradation within this compartment or upon trafficking to the lysosomes. However, similar cytosolic entrapment by forming MVBs has also been implicated in unconventional secretion, when the LEs/MVBs fused with the plasma membrane and release the ILVs, along their resident cargo, into the extracellular milieu as small extracellular vesicles (small EVs) or “exosomes” [24]. This process shares with e-MI the selective loading of cytosolic proteins, RNAs, and other molecules into ILVs, although in this case they are destined to be extracellularly released. Because degradative e-MI and MVB-mediated unconventional secretion occur in LEs, the convergence to these organelles seems to ensure some level of coordination between the two processes. For example, as described in previous sections, recent work points to the physiological importance of this secretory pathway in response to the progressive decline in e-MI and lysosomal function that occurs during aging [145]. The reduction in e-MI is accompanied by a compensatory increase in the secretion of the undigested material, indicating a shift in the function of MVBs formed via e-MI from a primarily degradative role to a secretory one [145]. In addition, as already described, the inhibition of CMA by acetylated TAU results in the rerouting of substrates into MVBs via HSPA8/HSC70-dependent e-MI, which are subsequently released outside the cell via small EVs or exosomes [152]. These emerging interactions between e-MI and secretion mechanisms reflect the growing number of examples in which disruptions in lysosomal proteolysis or acidification elicit a corresponding increase in the exocytosis via LEs/MVB intermediates, presumably as a final attempt by cells and tissues to maintain proteostasis during age-related lysosomal dysfunction [201–203].

Pathways employing the ATG8 protein conjugation machinery at the limiting membrane of the LEs utilize membrane dynamics reminiscent of MI-autophagy. Recent work uncovered that ATG8 proteins associate to subdomains of the limiting membrane of LEs/MVBs via a process termed conjugation of ATG8 to single membranes (CASM), which includes pathways such as LC3-associated phagocytosis (LAP) and LC3-associated endocytosis (LANDO) [204,205]. As described in previous sections, conjugation of ATG8 proteins to the MVB limiting membrane can coordinate the sorting into ILVs of cytosolic cargo, such as SQSTM1/p62, in the case of starvation-induced e-MI [25,128] (Table 2) or of lysosomal proteins, such as MCOLN1/TRPML1 (mucolipin TRP cation channel 1), in the case of ATG and ESCRT-dependent l-MI-lysophagy (Table 1) [25,128]. Since this ultimately results in the degradation of cargo in LEs, they are categorized as degradative MI-autophagy as discussed above. While certain CASM-driven processes culminate in lysosomal degradation, the incorporation of lipid-conjugated ATG8 proteins into ILVs is implicated in type III UPS. In particular, LC3 protein-positive ILVs can be released extracellularly as small EVs through a pathway termed LC3-dependent EV loading and secretion (LDELS) [206]. Indeed, ATG8 proteins are highly enriched within a subset of EVs and play critical a role in the loading of specific cargo into these vesicles, including RNA-binding proteins (RBPs) and small non-coding RNAs [206]. Since cargoes of the LDELS pathway are not degraded, this pathway is not formally classified as a type of MI-autophagy [207].

Similarly to other processes involving CASM, LDELS requires multiple core components of the ATG8 protein conjugation machinery, including ATG3, ATG7, and ATG12 (Table 3). Accordingly, cells lacking any of these *ATG* genes produce fewer small EVs and exhibit reduced EV-mediated secretion of multiple RBPs and small RNAs. In contrast, LDELS is independent of ATG proteins required for other steps of canonical macroautophagy, including RB1CC1/FIP200 and ATG14. Remarkably, CASM involves the conjugation of PS to LC3 proteins rather than PE, which is required for canonical autophagy [205,208]. Because PS is enriched in the membranes of small EVs [209], an important unanswered question is whether PS-conjugated LC3 proteins direct cargo into secretory pathways such as LDELS instead of classical degradative autophagy pathways.

Intralumenal budding during LDELS also functionally requires SMPD3/nSMase2 (sphingomyelin phosphodiesterase 3)-dependent production of ceramides at the limiting membrane of MVBs [210]. Furthermore, ATG8 proteins biochemically interact with NSMAF/FAN (neutral sphingomyelinase activation associated factor), which is required to activate this ESCRT-independent intralumenal budding pathway characterizing LDELS. Nevertheless, specific ESCRT components, including PDCD6IP/ALIX and HGS/HRS have been shown to functionally contribute to EV-dependent secretion of specific LDELS cargoes, such as TFRC (transferrin receptor) [211]. Overall, these results broach the hypothesis that various LDELS cargoes may employ substrate-specific mechanisms of ILV formation that are ESCRT-dependent and -independent. Furthermore, the extracellular release of LC3-positive small EVs requires the GTPase RAB27A [206,211], suggesting a pathway of exocytosis distinct from e-MI [145].

### MI-autophagy-related pathways in plants: ER-body and anthocyanin synthesis

The epidermal cell layer (aleurone layer) of the maize endosperm contains vacuoles that accumulate seed storage proteins critical for seed germination and seedling growth. Some of these storage proteins, specifically prolamins, are highly hydrophobic and form protein accretions inside the ER, termed ER protein bodies. During endosperm development, ER protein bodies are engulphed by the vacuoles present in aleurone cells and remain as stable vacuolar inclusions in the mature seed [212,213]. The vacuolar delivery of ER protein bodies represents a modality of v-MI-ERphagy and it does not depend on the canonical ATG machinery, as it proceeds normally in the maize *atg12* mutant endosperm (Table 3) [214].

Another form of MI-related pathway has been observed in some cell types and species that accumulate vacuolar anthocyanins, the flavonoid pigments that give grapes and cherries their characteristic red and purple colors. These flavonoids are synthesized in the cytoplasm and their biophysical and self-aggregation properties largely depend on decorations on their backbone. In *Arabidopsis thaliana*, anthocyanins derived from cyanidin-3-*O-*glucosyde are prone to form aggregates that are directly engulfed by the vacuolar membrane via a process akin to MI-autophagy; many other plant species have also been reported to accumulate densely packed anthocyanin vacuolar inclusions (AVIs) [215]. *Arabidopsis* anthocyanidins are not glycosylated at the 5-*O* position in the *5gt* (*UDP-glucose:cyanidin 5-O-glucosyltransferase*) mutant and they are accumulated in an acylated form as droplets in the cytoplasm. These anthocyanin-containing droplets bind the vacuolar membrane and are internalized into the vacuole through a v-MI-autophagy-like mechanism [200]. The formation of AVIs in *A. thaliana* does not require ATG5, a core component of macroautophagy [200], providing yet another example of an MI-related pathway independent of the core ATG machinery.

## Conclusions and perspectives

As highlighted in this review, the recent growth in number of studies interested in MI-autophagy is unveiling a previously unknown level of complexity on the molecular requirement for MI-autophagy. Besides the number of variants or subtypes, each MI-autophagy pathway shows different molecular requirements and can vary depending on MI-autophagy-inducing conditions and the organism. Nevertheless, there are however some common emerging themes such as for example the fact that the ESCRT complexes are required for all e-MI pathways and most of the v-MI- and l-MI-autophagic process in all eukaryotic cells with some exceptions, e.g., ATG-dependent v-MI-lipophagy and v-MI-chlorophagy. However, the ESCRTs requirement in v-MI and l-MI is something that has only recently been described [19,102,112], as such, many old reports did not examine ESCRTs, and some cases, ESCRT dependency has been hard to examine experimentally, especially in plants. The involvemenet of the ATG machinery is diverse and different among MI-autophagic pathways. The MI-pathways depending on core ATG proteins, e.g., v-MI-pexophagy, ATG-dependent v-MI-nucleophagy, and ATG-dependent v-MI-chlorophagy, exhibit ATG8 protein-positive structures formed during each v-MI-autophagy and seemed to be conserved from yeast to plants. The molecular function of ESCRTs in membrane deformation, i.e., curvature and fission, is well-established [216]. Specific ATG components such as ATG8 proteins and Atg18 appear to also have membrane fusion and scission activity at the vacuole during v-MI-pexophagy [36,41]. Therefore, a speculative notion is that MI-autophagy has evolved to employ ESCRT and/or ATG depending on and adapting to each MI-autophagic pathway and its induction conditions, possibly also in coordination with the type of macroautophagy pathway take place at the same time. In comparison to macroautophagy, our understanding of MI-autophagy is still in its infancy. Future researches addressing some of the open questions highlighted in the review will be critical to uncover the mechanisms and function of the different MI-autophagic pathways.

Cargo recognition in MI-autophagy involves a variety of mechanisms, depending in part on the type of cargo, i.e., protein vs. organelle, and on the lytic compartment engaged, i.e., vacuole, endosomes, or lysosomes. Although the identification of receptors and adaptors for e-MI is still in its beginnings, MI-ERphagy appears to be the most advanced in terms of receptor characterization. For protein-protein recognition, some ESCRT components and chaperones, such as HSPA8/HSC70, play a prominent role. Future efforts should aim to identify additional e-MI receptors and to elucidate the triage mechanisms among different types of MI-autophagy when they share receptors or adaptors.

As in the case of macroautophagy and CMA, MI-autophagy can be constitutively active but is also upregulated in response to different stimuli. Some of these stimuli are shared with other autophagic pathways, underscoring the need for better temporal dissection of the activation kinetics of each pathway and the signaling mechanisms involved.

While the physiological role of a few MI-autophagy pathways has been established, the overall pathophysiological relevance of MI-autophagy remains poorly understood. A major limitation in this regard is the small number of in vivo experimental interventions capable of selectively inhibiting one type of MI-autophagy without affecting the others. Identification of unique molecular components for each MI-autophagy pathway, and their selective genetic or pharmacological targeting, should help overcome this limitation.

## Abbreviations

aru: arouser; ATG: autophagy related; ATM: ATM serine/threonine kinase; ATP2A/SERCA: ATPase sarcoplasmic/endoplasmic reticulum Ca2+ transporting; ATR: ATR checkpoint kinase; AVI: anthocyanin vacuolar inclusion; BAG6: BAG cochaperone 6; BECN1: beclin 1; CALCOCO2: calcium binding and coiled-coil domain 2; CASM: conjugation of ATG8 to single membranes; CCPG1: cell cycle progression 1; cGAMP: cyclic GMP-AMP; CGAS: cyclic GMP-AMP synthase; CHEK1/Chk1: checkpoint kinase 1; CHMP: charged multivesicular body protein; CLEM: correlative light electron microscopy; CMA: chaperone-mediated autophagy; COL1A2: collagen type I alpha 2 chain; Cot1: cobalt toxicity 1; CPA: cyclopiazonic acid; Dpp1: diacylglycerol pyrophosphate phosphatase 1; DNAJC5: DnaJ heat shock protein family (HSP40) member C5; DNM1L: dynamin 1 like; DO: degradative organelle; dsDNA: double-stranded DNA; EEA1: early endosome antigen 1; EE: early endosome; e-MI: endosomal microautophagy; EPS8: EGFR pathway substrate 8, signaling adaptor; ER: endoplasmic reticulum; ERES: endoplasmic reticulum exit sites; ESCRT: endosomal sorting complex required for transport; EV: extracellular vesicle; Fet5: ferrous transport 5; Fth1: FTR1 Homolog 1; GABARAP: GABA type A receptor-associated protein; GAPDH: glyceraldehyde-3-phosphate dehydrogenase; Gem1: GTPase EF-hand protein of mitochondria; Hfl1: has fused lysosomes 1; HGS: hepatocyte growth factor-regulated tyrosine kinase substrate; HOPS: homotypic fusion and protein sorting; HSPA8/HSC70: heat shock protein family A (Hsp70) member 8; HSP: hereditary spastic paraplegia; MAPK/JNK: mitogen-activated protein kinase; MTORC1: mechanistic target of rapamycin complex 1; IKBKE: inhibitor of nuclear factor kappa B kinase subunit epsilon; ILV: intralumenal vesicle; IRF3: interferon regulatory factor 3; ITCH, itchy E3 ubiquitin protein ligase; LDELS: LC3-dependent EV loading and secretion; LAMP: lysosomal associated membrane protein; LANDO: LC3-associated endocytosis; LAP: LC3-associated phagocytosis; LAPTM4A: lysosomal protein transmembrane 4 alpha; LDs: lipid droplets; LE: late endosome; LIR: LC3-interacting region; l-MI: lysosomal microautophagy; MAP1LC3/LC3: microtubule associated protein 1 light chain 3; MAPT/TAU: microtubule associated protein tau; MCOLN1/TRPML1: mucolipin TRP cation channel 1; MDV: mitochondrial-derived vesicle; MI-autophagy: microautophagy; MIPA: micropexophagic membrane apparatus; MLV: murine leukemia virus; MVB: multivesicular body; NBR1: NBR1 autophagy cargo receptor; NCOA4: nuclear receptor coactivator 4; Ncr1: Niemann-Pick type C Related 1; NEDD4L: NEDD4 like E3 ubiquitin protein ligase; NFKB: nuclear factor kappa B; NSF: N-ethylmaleimide sensitive factor; NSMAF: neutral sphingomyelinase activation associated factor; NVJ: nucleus-vacuole junction; PAMP: pathogen-associated molecular pattern; PDCD6IP/ALIX: programmed cell death 6 interacting protein; PDH: pyruvate dehydrogenase; PE: phosphatidylethanolamine; Pho8: phosphate metabolism 8; PRKCI/PKCλ/ι: protein kinase C iota; PIK3C3: phosphatidylinositol 3-kinase catalytic subunit type 3; PMN: piecemeal MI-autophagy of the nucleus; PRKN: parkin RBR E3 ubiquitin protein ligase; PTP4A2/PRL2: protein tyrosine phosphatase 4A2; PRR: pattern recognition receptor; PS: phosphatidylserine; PtdIns3P: phosphatidylinositol-3-phosphate; PtdIns(3,5)P_2_: phosphatidylinositol-3,5-bisphosphate; PUB4: plant U-box4; RALA: RAS like proto-oncogene A; RB1CC1: RB1 inducible coiled-coil 1; RBP: RNA -binding protein; RE: recycling endosome; RETREG1: reticulophagy regulator 1; RHOT1: ras homolog family member T1; RNF152: ring finger protein 152; ROS, reactive oxygen species; RTN3L: reticulon 3 L; SAR: selective autophagy receptor; SCAMP3: secretory carrier membrane protein 3; SEC62: SEC62 preprotein translocation factor; SERINC5: serine incorporator 5; SGT: small glutamine rich tetratricopeptide repeat co-chaperone; SMPD3: sphingomyelin phosphodiesterase 3; SNARE: soluble NSF attachment protein receptor; SNCA: synuclein alpha; SP1: suppressor of ppi1 locus; SQSTM1: sequestosome 1; Ssh4: suppressor of Shr3 deletion; STAM1: signal transducing adaptor molecule 1; STK38: serine/threonine kinase 38; STING1: stimulator of interferon response cGAMP interactor 1; STX17: syntaxin 17; TAX1BP1, Tax1 binding protein 1; TBK1: TANK binding kinase 1; TEM: transmission electron microscopy; TFRC: transferrin receptor; TGN: trans-Golgi network; TOLLIP: toll interacting protein; TOC: translocon at the outer envelope membrane of chloroplasts; TOMM: translocase of outer mitochondrial membrane; TOMM20: translocase of outer mitochondrial membrane 20; TORC1: target of rapamycin complex 1; TSG101: tumor susceptibility 101; UBAP1: ubiquitin associated protein 1; ULK1, unc-51 like autophagy activating kinase 1; UPR: unfolded protein response; UPS: unconventional protein secretion; UVB: ultraviolet-B; VAMP8, vesicle associated membrane protein 8; Vba4: vacuolar basic amino acid transporter 4; v-MI: vacuolar microautophagy; Vph1: vacuolar pH 1; VPS: vacuolar protein sorting; Ycf1: yeast cadmium factor 1; Ypg2: yeast PQ-loop protein 2; Ypq1: yeast PQ-loop protein 1; WT: wild-type; Zrc1: zinc resistance conferring 3; Zrt3: zinc-regulated transporter 3; 5gt: UDP-glucose:cyanidin 5-O-glucosyltransferase

## Data Availability

Data sharing is not applicable to this article as no data were created or analyzed.
